# Unveiling the Unusual Mn(CO)_3_ Migration in a Manganese Cyclohexenyl Complex by DFT Computations

**DOI:** 10.3390/molecules29122945

**Published:** 2024-06-20

**Authors:** Guangchao Liang, Min Zhang

**Affiliations:** 1 Academy of Advanced Interdisciplinary Research, Xidian University, Xi’an 710071, China; 2 Department of Pharmacy, School of Medicine, Xi’an International University, Xi’an 710077, China; zhangmin01@xaiu.edu.cn

**Keywords:** agostic interaction, manganese complex, density functional theory, AIM analysis, hapticity

## Abstract

Homogeneous catalysis involving a transition metal agostic interaction (TM^…^H^…^C) is an attractive strategy for C–H bond activation, in which the transition metal agostic intermediates serve as the critical component. To investigate the roles of manganese agostic intermediates in the unusual migration of the Mn(CO)_3_ fragment in the (exo-phenyl)(η^3^-cyclohexenyl)manganese tricarbonyl [(Ph)(η^3^-C_6_H_8_)Mn(CO)_3_] (complex **1**) under the protonation of tetrafluoroboric acid–diethyl ether (HBF_4_.Et_2_O), a comprehensive density functional theory (DFT) theoretical study was performed. The computational results showed that formation of the [(cyclohex-3-enyl)-η^6^-benzene]manganese tricarbonyl complex [(C_6_H_9_)(η^6^-Ph)Mn(CO)_3_^+^][BF_4_] (complex **2**) was achieved via a series of mono-agostic and di-agostic intermediates. The overall rate-limiting step for this unusual migration of the Mn(CO)_3_ fragment is the formation of the di-agostic (η^2^-phenyl)manganese complex **8** (**4** → **5** → **8**) with a Gibbs barrier of 15.4 kcal mol^−1^. The agostic intermediates with TM^…^H^…^C agostic interactions were well-characterized by geometry parameters, Atoms-In-Molecules (AIM) analyses, and the Natural Adaptive Orbitals (NAdOs). The located pathways in the current study successfully explained the experimental observations, and the findings on the TM^…^H^…^C agostic interaction provided a new aspect of the catalytic reaction with the manganese complex.

## 1. Introduction

**Scheme 1:**
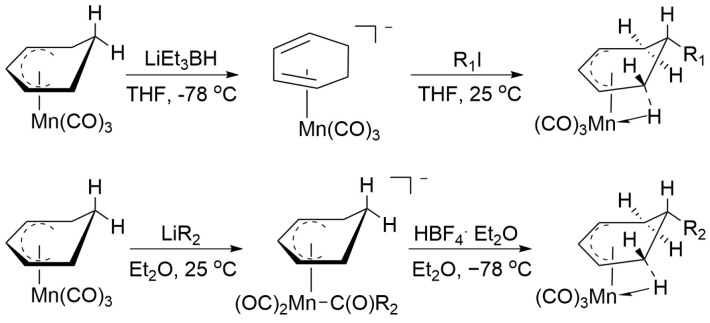
Strategies to synthesize substituted cyclohexenylmanganese agostic complexes.

Manganese tricarbonyl complexes are well known for their remarkable activities in homogeneous catalysis, and considerable efforts have been devoted to the design and synthesis of novel manganese tricarbonyl catalysts in recent decades [1,2,3,4]. The notion of the transition metal agostic interaction (TM^…^H^…^C) was first introduced by Brookhart and Green after the observations of the unique three-center two-electron TM-H-C bond in several transition metal complexes [5,6,7], and now the concept of agostic interactions, TM^…^H^…^C, in homogeneous catalysis is well established [8,9,10]. Two different strategies (Scheme 1) have been developed to synthesize the exo-substituted η^3^-cyclohexenylmanganese tricarbonyl complexes with the Mn^…^H^…^C agostic bond. In the first method, the (η^4^-cyclohexadienyl)manganese tricarbonyl anion species generated from hydride reduction in (η^5^-cyclohexadienyl)manganese tricarbonyl reacts with halogenated hydrocarbon (CH_3_I, R_1_ = CH_3_) to yield the target complex. Moreover, the protonation of the (η^4^-cyclohexadienyl)manganese tricarbonyl anion with water could generate the unsubstituted cyclohexenylmanganese tricarbonyl complexes (R_1_ = H) with an Mn^…^H^…^C agostic bond (top, Scheme 1) [5,6]. In the second method, the nucleophilic reaction between the (η^5^-cyclohexadienyl)manganese tricarbonyl and organolithium reagents (LiR_2_, R_2_ = Me, or Ph) preformed the (η^5^-cyclohexadienyl)manganese anion species. A following protonation with weak acid yields the target exo-substituted cyclohexenylmanganese tricarbonyl complexes (bottom, Scheme 1) [11,12,13].

**Scheme 2:**
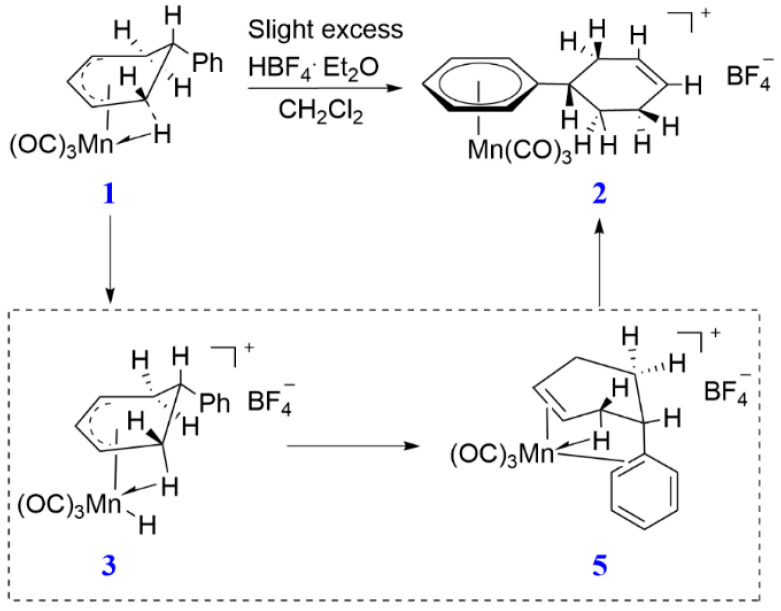
Migration of Mn(CO)_3_ from cyclohexenyl to benzene and the proposed intermediates.

When a slight excess of tetrafluoroboric acid–diethyl ether (HBF_4_^.^Et_2_O) was added to (exo-phenyl)(η^3^-cyclohexenyl)manganese tricarbonyl [(Ph)(η^3^-C_6_H_8_)Mn(CO)_3_] (complex **1**, Scheme 2), the protonation induced an unusual migration of the Mn(CO)_3_ group from the cyclohexenyl ring to the phenyl group, forming an unexpected product—[(cyclohex-3-enyl)-η^6^-benzene]manganese tricarbonyl complex [(C_6_H_9_)(η^6^-Ph)Mn(CO)_3_^+^][BF_4_] (complex **2**, Scheme 2) [12]. The η^3^ Mn-hydride species (complex **3**, Scheme 2) and (η^2^-cyclohexenyl)(η^2^-phenyl) complex (complex **5**, Scheme 2) have been proposed as the key intermediates in the migration of the Mn(CO)_3_ fragment [12]. In the previously proposed pathway, the protonation of (exo-phenyl)cyclohexenylmanganese tricarbonyl complex [(Ph)(η^3^-C_6_H_8_)Mn(CO)_3_] (complex **1**, Scheme 2) generated the η^3^ Mn-hydride species (complex **3**, Scheme 2), followed by the H transfer to terminal C atom of the η^3^-allyl unit to form the η^2^-cyclohexenyl species with one agostic bond. Then, the breaking of the Mn^…^H^…^C agostic bond yielded the (η^2^-cyclohexenyl)(η^2^-phenyl) complex (complex **5**, Scheme 2), which went through multiple arene ring-slip processes and finally led to the formation of the η^6^-benzene complex [(C_6_H_9_)(η^6^-Ph)Mn(CO)_3_^+^][BF_4_] (complex **2**, Scheme 2) [12]. However, the role of the agostic intermediate during the migration pathway has not been well described [14]. In addition, the H transfer of the η^3^ Mn hydride species (complex **3**, Scheme 2) to the terminal C atom of the η^3^-allyl moiety could potentially generate a η^2^-cyclohexenyl complex with two agostic bonds (one previously existed, and one was later formed from the terminal C atom with transferred H). Additionally, the agostic bond was also destroyed during the η^2^-arene to η^4^-arene ring-slip process. Comparisons of the relative positions of the double bond in the cyclohexenyl, phenyl group, and the agostic bond with Mn(CO)_3_ fragment suggest that the η^4^-phenyl complex with one agostic bond might be more favorable compared to the η^4^-phenyl complex with η^2^-cyclohexenyl group. Furthermore, the existence and roles of the η^2^-phenyl complexes with one agostic bond or two agostic bonds have not been addressed so far.

**Scheme 3:**
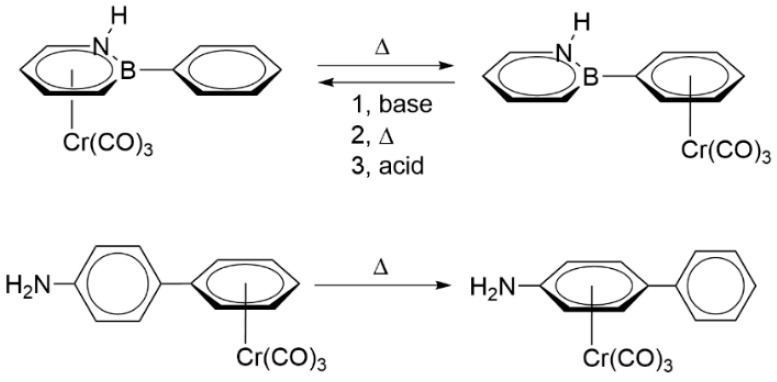
Haptotropic migration of Cr(CO)_3_ group between aromatic rings.

The previous studies on the haptotropic migration of the Cr(CO)_3_ group between the aromatic rings (Scheme 3) suggested that the Cr(CO)_3_ fragment migrates from the relatively electron-deficient aromatic ring to the electron-rich aromatic ring, and that conformational effects could inhibit the migration [15,16]. Experimental and theoretical studies have shown that the degree of ring coplanarity typically affects the Gibbs barrier to the migration of Cr(CO)_3_ fragment, and the possible participation of the ortho N-substituent has been verified by the comparison between the azaborine chromium tricarbonyl complex [(η^6^-C_4_BNH_5_)-(C_6_H_5_)Cr(CO)_3_] and para-aminobiphenyl chromium tricarbonyl complex [(η^6^-C_6_H_5_)(C_6_H_4_-4-NH_2_)]Cr(CO)_3_] [15,16,17,18,19]. As for (exo-phenyl)(η^3^-cyclohexenyl)manganese tricarbonyl [(Ph)(η^3^-C_6_H_8_)Mn(CO)_3_] (complex **1**, Scheme 2), it is not clear whether the protonation-induced migration of the Mn(CO)_3_ group follows the similar intramolecular inter-ring haptotropic rearrangement as observed in the Cr(CO)_3_ complexes. In addition, the conformational effects resulting from the possible agostic interaction could also promote the migration of Mn(CO)_3_ group from the relatively electron-deficient cyclohexenyl group to the electron-rich phenyl group, which also needs to be addressed [14].

In this paper, the density functional theory (DFT) computations were performed to investigate the migration mechanism of Mn(CO)_3_ from the cyclohexenyl ring to the phenyl group of (exo-phenyl)(η^3^-cyclohexenyl)manganese tricarbonyl [(Ph)(η^3^-C_6_H_8_)Mn(CO)_3_] (complex **1**, Scheme 2) under the protonation of HBF_4_^.^Et_2_O. The possible participation of the above proposed Mn^…^H^…^C agostic intermediates during the migration was examined, and the relative strength of the Mn^…^H^…^C agostic interaction was also evaluated by the Atoms-In-Molecules (AIM) analyses and the Natural Adaptive Orbitals (NAdOs).

## 2. Results and Discussion

To verify the reliability of the above computational method, the gas-phase PBEPBE/BS1-Auto optimized structure of [(cyclohex-3-enyl)-η^6^-benzene]manganese tricarbonyl complex **2**, [(C_6_H_9_)(η^6^-Ph)Mn(CO)_3_^+^][BF_4_], was compared with its reported X-ray structure (CSD entry: YUBXOI) [12] (Tables S1 and S2). A reasonable root-mean-square deviation (RMSD) of 0.2105 Å was obtained, with the crystal packing of CO groups causing the largest deviation. The additional comparisons from the gas-phase optimization of PBE-D3(BJ)/BS1-Auto and PBE-D3(BJ)/BS2-Auto were also performed, and slight improvements in RMSDs were observed. To further confirm the accuracy of the gas-phase optimization, the Gibbs free energies computed from gas-phase PBE/BS1-Auto and PBE-D3(BJ)/BS1-Auto were compared. An acceptable mean absolute deviation (MAD) of 1.64 (Table S3) and an excellent linear fitting (R^2^ = 0.9908, Figure S1) were presented. To reasonably address the effect of polarization functions of hydrogen atoms on the geometry optimization, the PBE/BS4-Auto optimized structures were matched with the PBE/BS1-Auto optimized ones, and significantly small values of RMSD (in Å) were obtained (Table S4). Additionally, no obvious differences in the electron density of bond critical point ρ_(BCP)_ from PBE/BS1-Auto optimized structures and PBE/BS4-Auto optimized ones could be observed (MSD = 0.004, MAD = 0.004) (Figures S3 and S4). These observations clearly demonstrated the suitability of current method in the geometry optimization, which is also consistent with our previous studies and the other reported work [20,21,22,23].

### 2.1. Pathways for the Mn(CO)_3_ Migration

**Figure 1:**
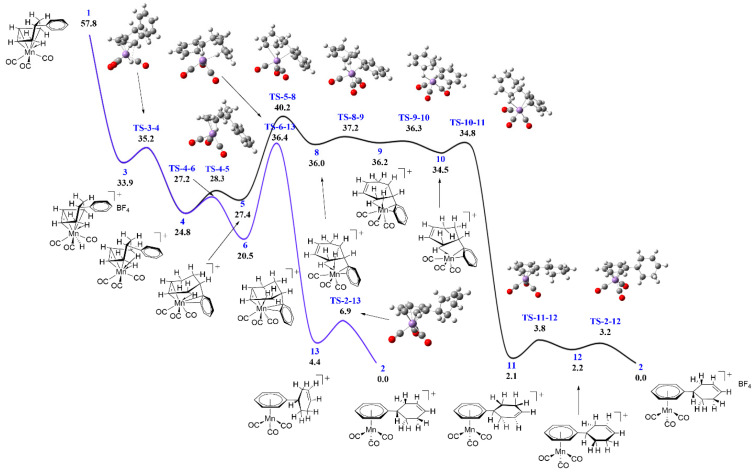
The free energy diagrams of the migration pathways from complex **1** to **2**. ΔG°/ΔG^‡^ from SMD(DCM)-PBE/BS2-Auto//PBE/BS1-Auto computations are given in kcal mol^−1^. All energies are relative to the complex **2**. The counterion BF_4_^−^ is omitted for clarity.

**Figure 2:**
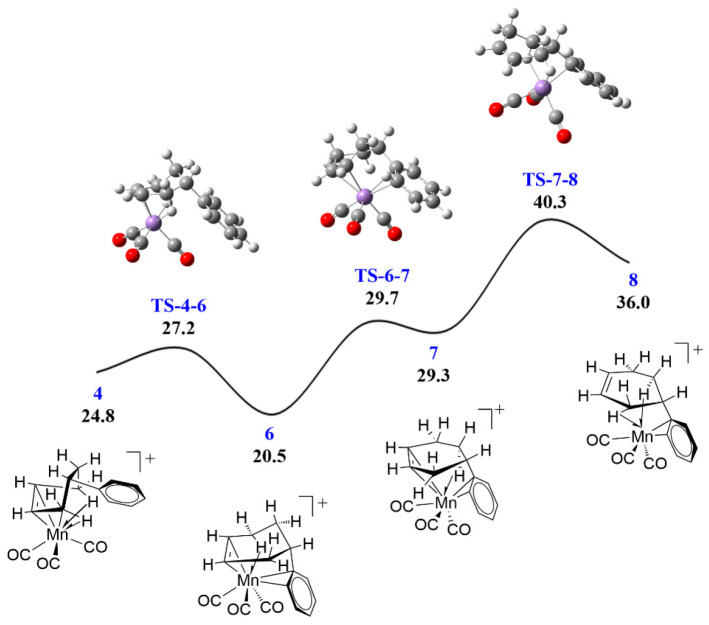
The free energy diagrams of the conversion from complex **4** to **8**. ΔG°/ΔG^‡^ from SMD(DCM)-PBE/BS2-Auto//PBE/BS1-Auto computations are given in kcal mol^−1^. All energies are relative to the complex **2**. The counterion BF_4_^−^ is omitted for clarity.

To gain insight into the unusual migration of the Mn(CO)_3_ fragment from the cyclohexenyl ring to the phenyl group (Scheme 2), the migration pathways were computationally investigated and are illustrated in Figure 1 and Figure 2. The protonation of (exo-phenyl)(η^3^-cyclohexenyl)manganese tricarbonyl [(Ph)(η^3^-C_6_H_8_)Mn(CO)_3_] (complex **1**, Scheme 2) by HBF_4_^.^Et_2_O in dichloromethane (DCM) initially generated the (η^3^-cyclohexenyl)Mn-hydride complex **3** (Figure 1), which is favorable by −23.9 kcal mol^−1^ (57.8 vs. 33.9 kcal mol^−1^, Figure 1). Once the (η^3^-cyclohexenyl)Mn-hydride complex **3** was formed, the following migration of the introduced hydride atom formed the di-agostic (η^2^-cyclohexenyl)manganese complex **4** with a Gibbs barrier of 1.3 kcal mol^−1^ (**3** → **TS-3-4** → **4**, Figure 1). Compared to the mono-agostic (η^3^-cyclohexenyl)Mn-hydride complex **3**, the di-agostic (η^2^-cyclohexenyl)manganese complex **4** is favorable by 9.1 kcal mol^−1^ (33.9 vs. 24.8 kcal mol^−1^, Figure 1). Due to the initially existing and the later formed (two) Mn-H-C agostic bonds in the di-agostic (η^2^-cyclohexenyl)manganese complex **4**, the subsequent breaking of these two Mn-H-C agostic units in complex **4** leads to two different mono-agostic (η^2^-phenyl)(η^2^-cyclohexenyl)manganese complex **5** (**4** → **5**) and complex **6** (**4** → **6**). The Gibbs barriers for the breaking of the initially existing Mn^…^H^…^C agostic bond in complex **4** to form complex **5** (Figure 1) and the breaking of later formed Mn-H-C agostic bond to form complex **6** (Figure 2) are 3.5 kcal mol^−1^ (**4** → **TS-4-5** → **5**, Figure 1) and 2.4 kcal mol^−1^ (**4** → **TS-4-6** → **6**, Figure 2), respectively. It is worth noting that the mono-agostic (η^2^-phenyl)(η^2^-cyclohexenyl)manganese complex **6** (20.5 kcal mol^−1^) is significantly lower in Gibbs free energy compared to its isomer complex **5** (27.4 kcal mol^−1^, Figure 1), and the steric effect from the methylene group is believed to be the main reason for the observed difference.

From the mono-agostic (η^2^-phenyl)(η^2^-cyclohexenyl)manganese complex **5**, the formation of the di-agostic (η^2^-phenyl)manganese complex **8** was located with a Gibbs barrier of 15.4 kcal mol^−1^ (24.8 kcal mol^−1^ for **4** vs. 40.2 kcal mol^−1^ for **TS-5-8**, Figure 1). Followed by the formation of the di-agostic (η^2^-phenyl)manganese complex **8**, the slipping of the η^2^-phenyl group formed another di-agostic (η^2^-phenyl)manganese complex **9** with a significant low Gibbs barrier of 2.0 kcal mol^−1^ (**8** → **9**, Figure 1). The mono-agostic (η^2^-phenyl)manganese complex **10** was then generated via the breaking of the endo-agostic bond in the di-agostic (η^2^-phenyl)manganese complex **9**, and the mono agostic η^2^-phenyl complex **10** was 1.7 kcal mol^−1^ more favorable compared to the di-agostic (η^2^-phenyl)manganese complex **9** (34.5 vs. 36.2 kcal mol^−1^, Figure 1). The breaking of the exo-agostic bond in the mono-agostic η^2^-phenyl complex **10** then generated the (η^6^-phenyl)manganese complex **11** (**10** → **11**, Figure 1), which was favorable by 32.4 kcal mol^−1^ (34.5 kcal mol^−1^ for **10** vs. 2.1 kcal mol^−1^ for **11**, Figure 1). To remain consistent with the reported X-ray structure of [(cyclohex-3-enyl)-η^6^-benzene]manganese tricarbonyl cation complex **2** (CSD entry: YUBXOI) [12], a two-step rotation of the cyclohexenyl groups was required from the (η^6^-phenyl)manganese complex **11** (**11** → **12** → **2**, Figure 1). As a straightforward C_ph_–C_cy_ single bond rotation, the expected low Gibbs barriers for the two-step rotation of 1.8 kcal mol^−1^ (**11** → **12**, Figure 1) and 1.0 kcal mol^−1^ (**12** → **2**, Figure 1) were obtained. The rate-limiting step in the first path (**4** → **5** → **8** → **9** → **10** → **11** → **12** → **2**, Figure 1) was the formation of the di-agostic (η^2^-phenyl)manganese complex **8** (**TS-5-8**, Figure 1) with an overall Gibbs barrier of 15.4 kcal mol^−1^ (24.8 kcal mol^−1^ for **4** vs. 40.2 kcal mol^−1^ for **TS-5-8**). A net Gibbs reaction energy of 57.8 kcal mol^−1^ in the exothermic migration of Mn(CO)_3_ group from the (exo-phenyl)(η^3^-cyclohexenyl)manganese tricarbonyl [(Ph)(η^3^-C_6_H_8_)Mn(CO)_3_] (complex **1**), forming the η^6^-benzene complex **2** [(C_6_H_9_)(η^6^-Ph)Mn(CO)_3_^+^][BF_4_], was then obtained. It is worth noting that the protonation-induced migration of the Mn(CO)_3_ group from (exo-phenyl)(η^3^-cyclohexenyl)manganese tricarbonyl [(Ph)(η^3^-C_6_H_8_)Mn(CO)_3_] (complex **1**) to form the η^6^-benzene complex **2** [(C_6_H_9_)(η^6^-Ph)Mn(CO)_3_^+^][BF_4_] was accomplished by a multi-step process, and a straightforward intramolecular inter-ring haptotropic rearrangement as presented in the Cr(CO)3 complexes [15,16,17,18,19] was not observed in the current case. A series of agostic intermediates (**3**, **4**, **5**, **7**, **8**, **9**, **10**) were involved in this protonation-induced migration of Mn(CO)_3_ group, promoting the migration of Mn(CO)_3_ group from the relatively electron-deficient cyclohexenyl group to the electron-rich phenyl group.

From the mono-agostic (η^2^-phenyl)(η^2^-cyclohexenyl)manganese complex **6,** two pathways were located leading to the formation of the di-agostic (η^2^-phenyl)manganese complex **8** (**6** → **7** → **8**, Figure 2) and the η^6^-benzene isomer complex **13**, [(C_6_H_9_)(η^6^-Ph)Mn(CO)_3_^+^][BF_4_] (**6** → **13**, Figure 1), respectively. In the first path, the vibration of the methylene group in the (η^2^-phenyl)(η^2^-cyclohexenyl)manganese complex **6** broke the hydride atom and introduced the Mn^…^H^…^C agostic bond, but formed a new (η^2^-phenyl)(η^2^-cyclohexenyl)manganese complex **7** (29.3 kcal mol^−1^, **6** → **7**, Figure 2) with the methylene group involved with the Mn-H-C agostic bond. A low Gibbs barrier of 9.2 kcal mol^−1^ was observed for the change in the Mn-H-C agostic bond (**6** → **7**, Figure 2). Next, the breaking of the Mn-C bonds between the η^2^-cyclohexenyl and manganese in complex **7** generated the di-agostic (η^2^-phenyl)manganese complex **8** with a Gibbs barrier of 10.9 kcal mol^−1^ (**7** → **8**, Figure 2). A slightly higher Gibbs barrier of 15.5 kcal mol^−1^ (24.8 kcal mol^−1^ for complex **4** vs. 40.3 kcal mol^−1^ for **TS-7-8**), compared with the 15.4 kcal mol^−1^ (24.8 kcal mol^−1^ for complex **4** vs. 40.2 kcal mol^−1^ for **TS-5-8**, Figure 1) for the rate-limiting step in the second path (**4** → **6** → **7** → **8**, Figure 2) was observed. Alternatively, in the second path (**6** → **13**, Figure 1), a single-step formation of another η^6^-benzene isomer complex **13** from the mono-agostic (η^2^-phenyl)(η^2^-cyclohexenyl)manganese complex **6** was also successfully located with a Gibbs barrier of 15.9 kcal mol^−1^ (**6** →**TS-6-13** → **13**, Figure 1). Once the η^6^-benzene isomer complex **13** was formed, a simple rotation of the cyclohexenyl group led to the final η^6^-benzene complex **2**, [(C_6_H_9_)(η^6^-Ph)Mn(CO)_3_^+^][BF_4_] with a low Gibbs barrier of 2.5 kcal mol^−1^ (**TS-2-13**, Figure 1).

### 2.2. Characterization of Agostic Complexes

The above established pathways on the unusual protonation-induced migration of the Mn(CO)_3_ fragment from the cyclohexenyl group to the phenyl group (Figure 1 and Figure 2) presented the following observations: (1) the overall rate-limiting step for this unusual migration is the formation of di-agostic (η^2^-phenyl)manganese complex **8** (**4** → **TS-4-5** → **5** → **TS-5-8** → **8**, Figure 1) with a Gibbs barrier of 15.4 kcal mol^−1^ (24.8 kcal mol^−1^ for **4** vs. 40.2 kcal mol^−1^ for **TS-5-8**); (2) the exothermic reaction from complex **3** to complex **2** is overall favorable by 33.9 kcal mol^−1^; and (3) the mono agostic complexes (**3**, **5**, **6**, **7,** and **10**) and di-agostic complexes (**4**, **8,** and **9**) served as the main intermediates in the above exothermic migration. To better understand the roles of these agostic complexes in the migration of Mn(CO)_3_, the agostic bonds in these intermediates were further computationally characterized and well analyzed.

Several well-known geometry parameters and bonding characters in the agostic complex include the following: (*i*) short TM-H distance (1.8–2.3 Å), (*ii*) small TM^…^H^…^C bond angle (90–140°), (*iii*) up field-shift agostic hydrogen, and (*iv*) low spin coupling J_CH_ (50–100 Hz) [7,24]. Studies of the orbital interactions in the Mn^…^H^…^C agostic bonding indicated that both the σ_(C-H)_ → *d*_(TM)_ and *d*_(TM)_ → σ*_(C-H)_ π-back donation interactions contributed to the Mn^…^H^…^C agostic bond [9,25]. DFT-computed agostic parameters of the mono-agostic complexes (**3**, **5**, **6**, **7** and **10**) and di-agostic complexes (**4**, **8** and **9**) are summarized in Table 1.

**Figure 3:**
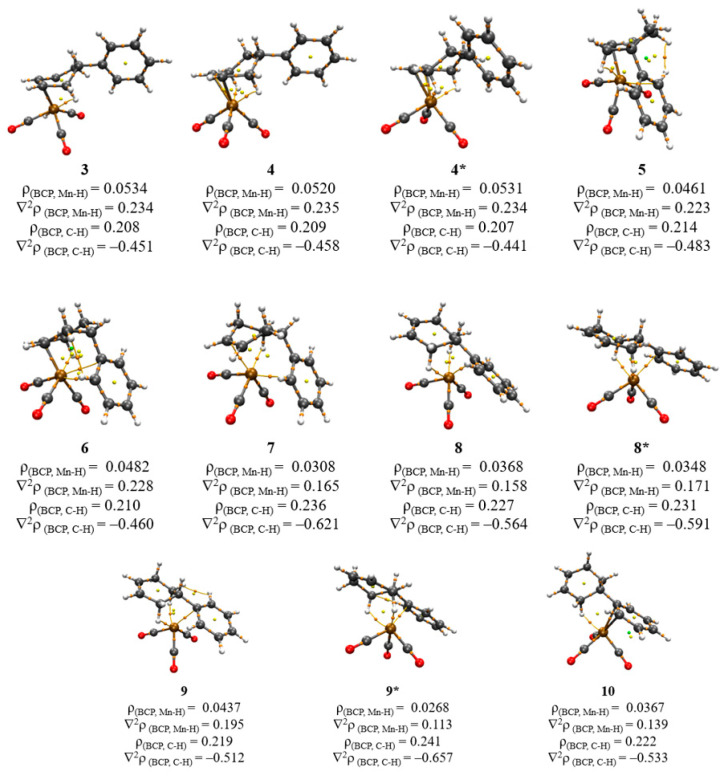
The AIM (Atoms-In-Molecules) analysis of the agostic complexes. The orange balls represent the BCP (bond critical point), the yellow balls represent RCP (ring critical point), the green balls represent CCP (cage critical point), and the bond paths are shown in orange. Atom color codes: C, gray; H, white; O, red; Mn, ochre. The electron densities of bond critical points [ρ_(BCP)_] and Laplacian of electron density (∇^2^ρ) are given in a.u. The asterisks represent the endo-agostic Mn^…^H^…^C unit in the di agostic complexes **4**, **8** and **9**. The counterion BF_4_^−^ is omitted for clarity. The asterisks represent the endo agostic Mn^…^H^…^C bond in the di agostic complexes **4**, **8** and **9**.

It is clear from Table 1 that all these complexes (**3**, **4**, **5**, **6**, **7**, **8**, **9** and **10**) fit the above well-known geometrical parameters of an agostic complex with the shortened Mn–H distance (1.813–2.085 Å), the prolonged C–H_(agostic)_ bond length (1.133–1.189 Å), and the small Mn^…^H^…^C bond angle (93.7–119.8°). The J_CH_ coupling constants in the Mn^…^H^…^C unit of these mono-agostic complexes (**3**, **5**, **6**, **7** and **10**) and di-agostic complexes (**4**, **8** and **9**) were about 53 Hz (averaged) lower than those J_CH_ couplings of the non-agostic ones. The high field agostic hydrogen atoms in the Mn^…^H^…^C agostic unit compared to the non-agostic hydrogen were also confirmed by the proton chemical shifts (by 6.9 ppm, averaged). The AIM (Atoms-In-Molecules) analyses of the Mn^…^H^…^C unit in the mono-agostic complexes (**3**, **5**, **6**, **7** and **10**) and di-agostic complexes (**4**, **8** and **9**) were presented in Figure 3. The relative strength of a Mn–H bond and a H–C bond in the Mn^…^H^…^C agostic unit could be measured by the calculated electron densities of bond critical points [ρ_(BCP)_] and the absolute value of the Laplacian of electron density (∇^2^ρ) (Figure 3), and a stronger chemical bond is characterized as a shorter bond distance and bigger Wiberg bond index, which could be demonstrated by the comparison of agostic complexes **3** and **5**. The Mn–H distance in agostic complexes **3** and **5** are 1.823 and 1.977 Å (Table 1), respectively, showing a stronger Mn–H bond in agostic complex **3** compared with complex **5**. It was also confirmed by the computed Wiberg bond index of complexes **3** and **5** (0.16 vs. 0.11, Table 1). The calculated electron densities of Mn–H bond critical points [ρ_(BCP)_] for agostic complexes **3** and **5** are 0.0534 and 0.0461 a.u. (Figure 3), and the related absolute value of the Laplacian of electron density (∇^2^ρ) are 0.234 and 0.223 a.u., respectively (Figure 3). It is worth noting that the endo Mn^…^H^…^C agostic unit in the di-agostic complex **9** (the 9*) had the longest Mn–H distance of 2.085 Å among all the agostic Mn–H bonds and had the shortest C–H bond of 1.133 Å (Table 1) among all the agostic C–H bonds. Consequently, the smallest value of electron density of Mn–H bond critical points [ρ_(BCP)_] (0.0268 a.u., Figure 3) and the smallest value of Laplacian of electron density (∇^2^ρ) (0.113 a.u., Figure 3) were observed in endo-agostic Mn-H-C unit of the di-agostic complex **9** (the 9*). In contrast to the weak agostic Mn–H bond, the strongest agostic C–H bond of 1.133 Å in the di-agostic complex **9** (the 9*) was verified by the biggest value of electron density of agostic C–H bond critical points [ρ_(BCP)_] (0.241 a.u., Figure 3) and the biggest absolute value of Laplacian of electron density (∇^2^ρ) (0.657 a.u., Figure 3). The AIM analyses of other agostic intermediates were also obtained and presented in Figure 3, confirming the existence of the Mn^…^H^…^C agostic interaction, which was consistent with previous reports [20,21].

**Figure 4:**
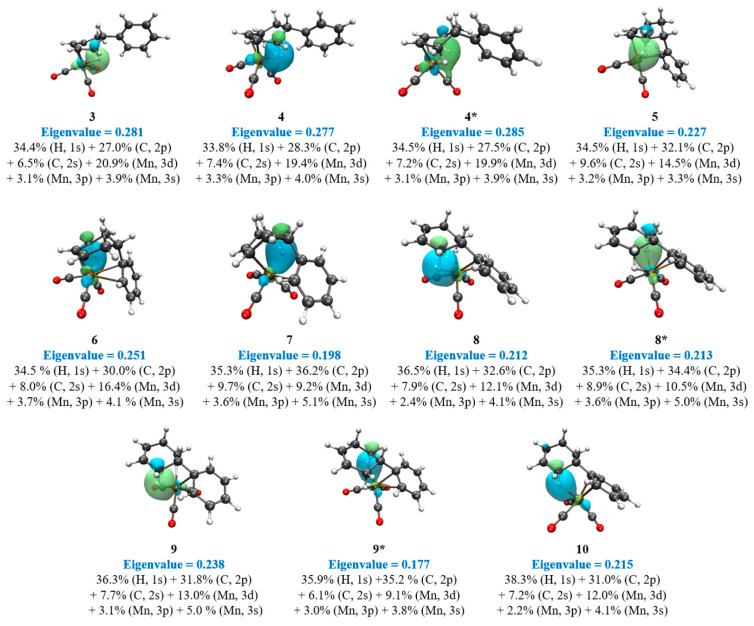
The NAdOs (natural adaptive orbitals, isovalue = 0.05) of the Mn^…^H^…^C agostic interactions. Atom color codes: C, gray; H, white; O, red; Mn, ochre. The asterisks represent the endo-agostic Mn^…^H^…^C bond in the di-agostic complexes **4**, **8** and **9**. The counterion BF_4_^−^ is omitted for clarity.

To visually evaluate the agostic interactions in mono-agostic complexes (**3**, **5**, **6**, **7** and **10**) and di-agostic complexes (**4**, **8** and **9**), the NAdOs (natural adaptive orbitals) of the Mn^…^H^…^C agostic interaction were introduced (Figure 4). Analyses of the NAdOs provided the following facts: (1) the eigenvalues of NAdOs of the Mn^…^H^…^C agostic unit in the complexes **3**, **4** and 4* are significantly bigger than others (0.281,0.277 and 0.285, respectively, Figure 4); (2) the smallest eigenvalue of 0.177 for agostic complex 9* is observed with the least contribution of 3*d*_(Mn)_ orbital into the NAdO of the Mn^…^H^…^C agostic unit (9.1%); (3) the highest contribution of 3d_(Mn)_ to the NAdO of the Mn^…^H^…^C agostic unit (20.9%) is observed in the mono agostic complex **3**; and (4) the contribution of 2*p*_(C)_ orbital to the NAdO of the Mn^…^H^…^C agostic unit in agostic complex 9* is remarkably higher than that of agostic complex **3** (35.2% vs. 27.0%, Figure 4). The higher contribution of the 3*d*_(Mn)_ orbital to the NAdOs of the Mn^…^H^…^C agostic unit in agostic complex **3** compared to complex 9* agrees with the relative stronger Mn–H bond in complex **3** than that of complex 9* (Table 1, Figure 3) [20]. Meanwhile, the higher contribution of 2*p*_(C)_ orbital to the NAdOs of the Mn^…^H^…^C agostic unit in agostic complex 9* compared to complex **3** is entirely consistent with the stronger agostic C–H bond in complex 9* than that of complex **3** (Table 1, Figure 3). Additionally, to investigate the role of Mn^…^H^…^C agostic interactions in the stabilization of Mn agostic intermediates, the second order perturbative energy, E^(2)^, was obtained from the NBO computation. NBO analyses showed that the interaction of the σ_(C-H)_ donor with the 3d*_(Mn)_ empty acceptor (σ_(C-H)_ → 3d*_(Mn)_) was the major contribution in Mn^…^H^…^C agostic interaction, and relative weak contribution from the back-donation of 3*d*_(Mn)_ donor to the σ*_(C-H)_ acceptor was also located (Table S5). Not surprisingly, the lowest estimated stabilization energy of the Mn^…^H^…^C agostic interaction via the computed E^(2)^ from the dominant σ_(C-H)_ → 3d*_(Mn)_ interaction in agostic complex 9* was observed (28.95 kcal mol^−1^, Table S5), which was notably lower than that of agostic complex **3** (63.63 kcal mol^−1^, Table S5).

## 3. Computational Methods

Gas-phase geometry optimizations were performed using the Gaussian 16 (Revision C 01) [26] package with PBE functional [27] (as PBEPBE in the Gaussian 16 package) and basis sets 1 (BS1). In BS1, the modified-LANL2DZ with the *f* polarization (modified-LANL2DZ(*f*)) [28,29,30] and the effective core potential (ECP, LANL2DZ) were utilized for the Mn atom, the 6-31G (*d’*) [31,32,33] basis sets were employed for the C, O, and H atoms, and the LANL2DZ(*d*, *p*) [34,35] with the related ECP (LANL2DZ) were used for the Si atom in the reference system TMS. Vibrational frequency computations were used to verify the natures of all stationary points, all located minima were confirmed with no imaginary frequency, and all located transition states were obtained with only one imaginary frequency. The IRC (intrinsic reaction coordinate) computations following the transition state were performed. Natural bond orbital (NBO) [36,37,38,39] analysis and Wiberg bond index [40,41] were performed with the Gaussian 16 integrated NBO program (NBO version 3). To address the solvation effect in dichloromethane (DCM), the PBEPBE/BS2 self-consistent reaction field (SCRF) single-point computations were performed with the solvation model based on density (SMD) [42]. The reported value solvation free energy of the proton (ΔG_H_^+^_,solv_ = −207.79 kcal mol^−1^) [43,44] and the experimental value of the gas-phase Gibbs free energy of a proton (ΔG_H+,gas_ = −6.28 kcal mol^−1^) [45] were used. In BS2, the Ahlrichs redefined Def2-TZVP [46,47] basis sets were utilized for H, C, O and Mn atoms. The counterion BF_4_^−^ was excluded from the computations. All computations were performed at 1 atm and 298.15 K, and the automatic density fitting approximation (via Auto keyword) [48,49] with pure spherical harmonic 5*d* and 7*f* functions were utilized. For comparison, the gas-phase optimizations using Grimme’s D3 [50] dispersion with Becke–Johnson damping [D3(BJ)] [51] (PBE-D3(BJ)/BS1-Auto and PBE-D3(BJ)/BS2-Auto) were also performed (see Supporting Information for the detailed comparisons). The Gibbs free energies from SMD(DCM)-PBE/BS2-Auto//PBE/BS1-Auto computations are presented in the main text, and the results from the PBE-D3(BJ)/BS1-Auto and PBE-D3(BJ)/BS2-Auto computations are presented in the Supporting Information. To reasonably evaluate the effect of polarization functions on hydrogen atoms, additional geometrical optimizations with BS4 (PBE/BS4-Auto) were performed. In BS4, the modified-LANL2DZ with the *f* polarization (modified-LANL2DZ(*f*)) [28,29,30] and the effective core potential (ECP, LANL2DZ) were utilized for the Mn atom, while the 6-31G (*d*, *p*) basis sets [31,32] were employed for the C, O, and H atoms.

The J_CH_ spin coupling computations were carried out using the Gauge-Independent Atomic Orbital (GIAO) [52,53,54] method with PBEPBE functional and basis set 3 (BS3) based on the gas-phase optimized structures. In BS3, LANL08(*f*) [29,55] and ECP (LANL2DZ) basis sets were employed for Mn atom, LANL08(*d*) [35,55] and related ECP (LANL2DZ) for Si, and the 6-311G++(3*df*, 3*pd*) [56,57] basis sets for other atoms (C, O, and H). All simulated proton chemical shifts were relative to the absolute shift of tetramethylsilane (TMS). The electron density of bond critical point [ρ_(BCP)_] based on Bader’s theory of atoms-in-molecules (AIM) [58,59,60] and natural adaptive orbitals (NAdOs) [61] were calculated by Multiwfn package (version 3.8) [62,63], and were then visualized by VMD package (version 1.9.3) [64,65].

## 4. Conclusions

The query on the experimentally observed unusual migration of the Mn(CO)_3_ fragment from the cyclohexenyl ring to the phenyl group in the protonated (exo-phenyl)(η^3^-cyclohexenyl)manganese tricarbonyl [(Ph)(η^3^-C_6_H_8_)Mn(CO)_3_] (complex **1**) was correctly addressed by density functional theory (DFT) computations. DFT computational results showed that formation of the final product, [(cyclohex-3-enyl)-η^6^-benzene]manganese tricarbonyl complex [(C_6_H_9_)(η^6^-Ph)Mn(CO)_3_^+^][BF_4_] (complex **2**), was accomplished via a series of mono-agostic intermediates (**3**, **5**, **6**, **7** and **10**) and di-agostic intermediates (**4**, **8** and **9**). The overall rate-limiting step for this unusual migration of the Mn(CO)_3_ fragment from the cyclohexenyl ring to the phenyl group is the formation of the di-agostic (η^2^-phenyl)manganese complex **8** (**4** → **5** → **8**) with a Gibbs barrier of 15.4 kcal mol^−1^. The Mn^…^H^…^C agostic interactions in this unusual migration were well characterized by geometry parameters, AIM (Atoms-In-Molecules) analyses, and the NAdOs (natural adaptive orbitals). The obtained diverse contributions of 3d_(Mn)_ orbital (20.9% for complex **3** vs. 9.1% for complex 9*) and 2p_(C)_ orbital (27.0% for complex **3** vs. 35.2% for complex 9*) to the NAdO of the Mn^…^H^…^C agostic unit were in agreement with the calculated electron densities of Mn–H bond critical points [ρ_(BCP)_]. Compared to the classical two-electron donor ligand, the bond strength of a Mn^…^H^…^C agostic bond is relatively weak, especially in the di-agostic intermediates, leading to a feasible and practicable C−H bond activation via the conversion from agostic Mn^…^H^…^C unit to a Mn–H bond. Furthermore, the catalytic activation of small molecules (such as CO_2_ and CH_4_) via transition metal complexes has usually been inhibited by the limited accessible area of metal site. The “opening” site in the mono-agostic and di-agostic intermediates made the nucleophilic attack of CO_2_ or CH_4_ on the Mn metal site accessible, thus achieving the catalytic activation of the small molecule. Insights gained in this study on the Mn^…^H^…^C agostic interactions provide a new aspect of catalytic reaction with Mn complex, resulting the design of novel and efficient Mn complex for homogeneous catalysis.

## Figures and Tables

**Table 1:** DFT-computed agostic parameters of the agostic complexes. Bond length of Mn-hydride in complex **3** is 1.546 Å, Wiberg bond index of Mn-hydride is 0.51, and ^1^H chemical shift is −8.7 ppm. The asterisks represent the endo agostic Mn^…^H^…^C bond in the di agostic complexes **4**, **8** and **9**. Non ^#^ represents the non-agostic C–H bond.

Complex	Mn-H (Å)	C-H (Å)		Mn-H-C (°)	J_C-H_ (Hz)		σ(H) (ppm)		Wiberg Bond Index		
		Agostic	Non^#^		Agostic	Non^#^	Agostic	Non^#^	Mn-H	Agostic	Non^#^
**3**	1.823	1.185	1.102	98.4	65.9	135.6	−7.4	2.5	0.16	0.65	0.85
**4**	1.826	1.184	1.103	98.4	67.4	133.2	−8.1	2.9	0.15	0.66	0.85
4^*^	1.813	1.189	1.103	99.0	63.5	130.5	−7.7	3.3	0.16	0.66	0.85
**5**	1.977	1.165	1.108	93.7	73.5	127.8	−2.5	4.4	0.11	0.72	0.83
**6**	1.897	1.173	1.104	99.3	69.3	128.0	−5.3	3.2	0.14	0.70	0.85
**7**	1.998	1.141	1.105	107.7	79.9	124.2	−4.5	2.9	0.09	0.79	0.86
**8**	1.946	1.152	1.106	110.2	74.7	122.9	0.6	3.7	0.10	0.75	0.86
8^*^	1.947	1.145	1.104	111.5	77.6	120.9	−2.5	2.8	0.10	0.77	0.88
**9**	1.862	1.164	1.107	110.5	69.1	121.7	−1.5	3.5	0.12	0.72	0.86
9^*^	2.085	1.133	1.102	111.7	84.7	120.4	0.5	3.1	0.07	0.81	0.88
**10**	1.949	1.159	1.106	119.8	71.9	122.1	−1.8	3.6	0.10	0.76	0.87

## Data Availability

Data are contained within the article and Supplementary Materials.

## Supplementary Materials

The following supporting information can be downloaded at: https://www.mdpi.com/article/10.3390/molecules29122945/s1, Table S1: The matched DFT optimized structure with reported X-ray crystal structure; Table S2: Selected bond lengths and angles for complex **2**; Table S3: Comparisons of the Gibbs free energies; Figure S1: Linear fitting; Table S4. The matched DFT optimized structure; Table S5: Comparisons of the Gibbs free energies; Figure S2: Linear fitting; Figure S3: The AIM analysis of complex **1**; Figure S4. Comparisons of the ρ_(BCP)_; Figure S5: The NAdOs of complex **1**; Table S6: DFT computed agostic parameters of the agostic complex **1**; Table S7: Donor and acceptor NBOs of the Mn-H-C agostic unit; Figures S6–S8: IRC plots; Table S8: Cartesian coordinates.
