# Supplementary material for: Unveiling the Unusual Mn(CO)3 Migration in a Manganese Cyclohexenyl Complex by DFT Computations
Source: Molecules. 2024 Jun 20;29(12):2945. doi: 10.3390/molecules29122945 (PMC11206625; doi:10.3390/molecules29122945)
Supplement: Supplementary file 1 [file molecules-29-02945-s001.zip › Supplementary File/SI-06-18-2024.pdf]

-- Supporting information --

Unveiling the Unusual $Mn(CO)_3$ Migration in a Manganese Cyclohexenyl Complex by DFT Computations

Guangchao Liang*[1] and Min Zhang [2]

[1] Academy of Advanced Interdisciplinary Research, Xidian University, Xi'an, Shaanxi 710071, P.R. China
[2] Department of Pharmacy, School of Medicine, Xi'an International University, Xi'an, Shaanxi 710077, P.R. China

Table of Contents

Table S1. The matched DFT optimized structure with reported X-ray crystal structure. S2

Table S2. Selected bond lengths (in Å) and angles (in °) for complex 2. .... S2

Table S3. Comparisons of the Gibbs free energies computed from gas-phase PBE/BS1-Auto and PBE-D3(BJ)/BS1-Auto. S3

Figure S1. Linear fitting between the gas-phase PBE/BS1-Auto computed Gibbs free energies and PBE-D3(BJ)/BS1-Auto computed Gibbs free energies. S3

Table S4. The matched DFT optimized structure. S4

Table S5. Comparisons of the Gibbs free energies computed from SMD(DCM)-PBE/BS2-Auto//PBE/BS1-Auto and Gibbs energies from SMD(DCM)-PBE/BS2-Auto//PBE/BS4-Auto. S5

Figure S2. Linear fitting between the Gibbs energies from SMD(DCM)-PBE/BS2-Auto//PBE/BS1-Auto and Gibbs energies from SMD(DCM)-PBE/BS2-Auto//PBE/BS4-Auto. S5

Figure S3. The AIM (Atoms-In-Molecules) analysis of complexes 1, 3, and 4. .... S6

Figure S4. Comparisons of the $\rho_{(BCP)}$ from PBE/BS1-Auto and PBE/BS4-Auto optimized structures S7

Figure S5. The NAdOs (natural adaptive orbitals, isovalue = 0.05) of the Mn-H-C agostic interaction in complex 1. .... S7

Table S6. DFT computed agostic parameters of the agostic complex 1. .... S8

Table S7. Donor and acceptor NBOs of the Mn-H-C agostic unit. S8

Figure S6. IRC plot for TS-4-6. .... S8

Figure S7. IRC plot for TS-7-8 .... S9

Figure S8. IRC plot for TS-4-13 .... S9

Table S8. Cartesian coordinates and computed energies (in Hartrees) of optimized structures S10

Table S1. The matched DFT optimized structure with reported X-ray crystal structure. Hydrogen atoms are omitted to calculate the RMSD (in Å) and X-ray crystal structures are presented in green (CSD entry: YUBXOI).

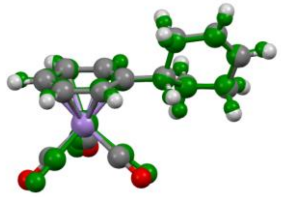	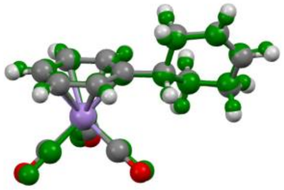	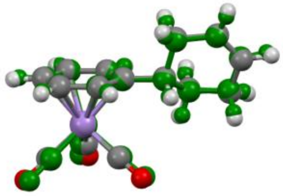
**Complex 2**<br>PBE/BS1-Auto<br>RMSD = 0.2105	**Complex 2**<br>PBE-D3(BJ)/<br>BS1-Auto<br>RMSD = 0.1657	**Complex 2**<br>PBE-D3(BJ)/<br>BS2-Auto<br>RMSD = 0.1387

BS1: modified-LANL2DZ(*f*) and the effective core potential (ECP, LANL2DZ) for Mn atom; 6-31G (*d'*) for all other atoms (C, O, and H). BS2: Ahlrichs redefined Def2-TZVP for H, C, O and Mn.

Table S2. Selected bond lengths (in Å) and angles (in °) for complex **2**.

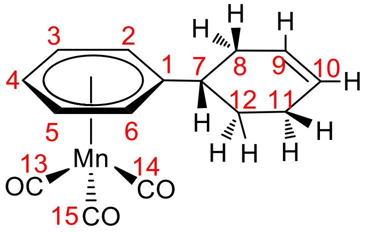

Parameter	Expt.	Compt.<br>PBE/BS1-Auto
Mn-C1	2.208	2.263
Mn-C2	2.197	2.217
Mn-C3	2.186	2.203
Mn-C4	2.168	2.205
Mn-C5	2.171	2.202
Mn-C6	2.188	2.208
Mn-C13	1.795	1.795
Mn-C14	1.813	1.793
Mn-C15	1.820	1.795
C1-C6	1.400	1.441
C1-C2	1.396	1.422
C2-C3	1.392	1.431
C3-C4	1.367	1.413
C4-C5	1.404	1.431
C5-C6	1.407	1.413

C1-C7	1.523	1.511
C7-C8	1.537	1.558
C7-C12	1.518	1.543
C1-C7-C8	108.4	108.6
C1-C7-C12	116.0	115.4
C13-Mn-C14	88.7	89.1
C13-Mn-C15	90.6	89.2
C14-Mn-C15	90.4	89.3
C3-Mn-C15	86.0	89.7
C5-Mn-C13	87.5	90.3
C1-Mn-C14	87.2	89.1

Table S3. Comparisons of the Gibbs free energies computed from gas-phase PBE/BS1-Auto and PBE-D3(BJ)/BS1-Auto.

**Species**	PBE/BS1-Auto (kcal/mol)	PBE-D3(BJ)/BS1-Auto (kcal/mol)
Complex 3	38.2	37.6
TS-3-4	39.4	38.8
Complex 4	17.7	17.0
TS-4-6	27.6	25.3
Complex 6	15.6	11.6
**MSD = –1.64, MAD = 1.64**		

MSD: mean signed deviation. MAD: mean absolute deviation. Complex **2** as the reference of 0.0 kcal/mol. BS1: modified-LANL2DZ(*f*) and the effective core potential (ECP, LANL2DZ) for Mn atom; 6-31G (*d'*) for all other atoms (C, O, and H).

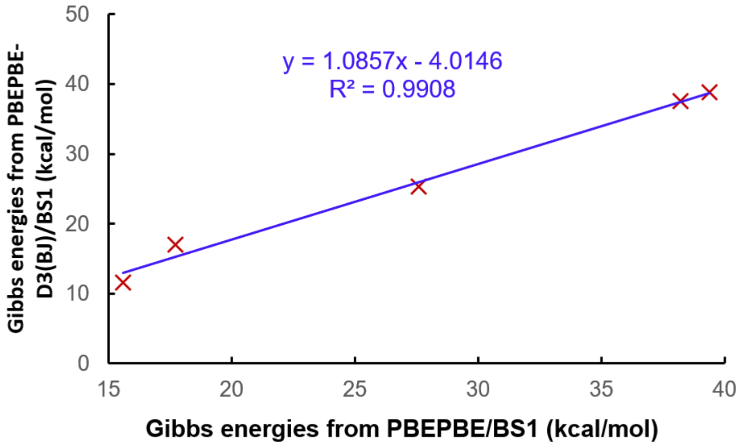

Figure S1. Linear fitting between the gas-phase PBE/BS1-Auto computed Gibbs free energies and PBE-D3(BJ)/BS1-Auto computed Gibbs free energies.

Table S4. The matched DFT optimized structure.

The gas-phase PBE/BS1-Auto optimized structures are presented in blue and the gas-phase PBE/BS4-Auto optimized structures are presented in red. All atoms are included to calculate the RMSD (in Å).

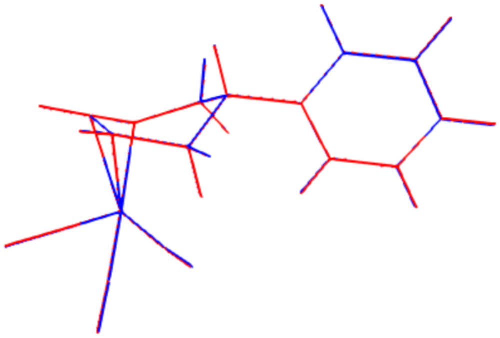 **Complex 1** RMSD = 0.0253	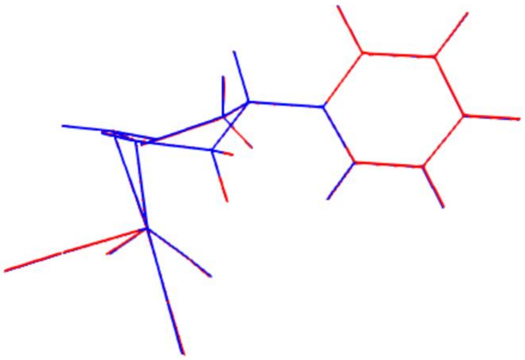 **Complex 3** RMSD = 0.0604
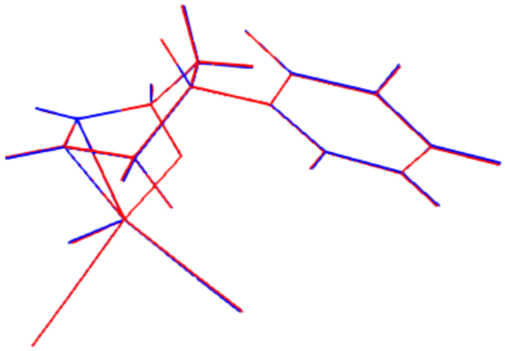 **Complex 4** RMSD = 0.0401	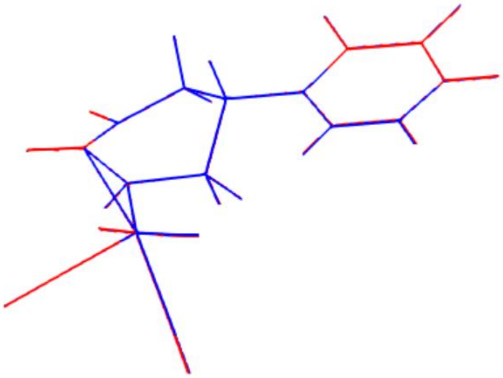 **TS-3-4** RMSD = 0.0229

BS1: modified-LANL2DZ(*f*) and the effective core potential (ECP, LANL2DZ) for Mn atom; 6-31G (*d'*) for all other atoms (C, O, and H). BS4: modified-LANL2DZ(*f*) and the effective core potential (ECP, LANL2DZ) for Mn atom; 6-31G (*d*,p) for all other atoms (C, O, and H).

Table S5. Comparisons of the Gibbs free energies computed from SMD(DCM)-PBE/BS2-Auto//PBE/BS1-Auto and Gibbs energies from SMD(DCM)-PBE/BS2-Auto//PBE/BS4-Auto.

**Species**	SMD(DCM)-PBE/BS2-Auto//PBE/BS1-Auto (kcal/mol)	SMD(DCM)-PBE/BS2-Auto//PBE/BS4-Auto (kcal/mol)
Complex 3	33.9	33.9
TS-3-4	35.2	35.4
Complex 4	24.8	24.7
TS-4-6	27.2	27.5
Complex 6	20.5	20.5
TS-4-5	28.3	28.0
Complex 5	27.4	28.0
**MSD = 0.10, MAD = 0.21**		

MSD: mean signed deviation. MAD: mean absolute deviation. Complex **2** as the reference of 0.0 kcal/mol. BS1: modified-LANL2DZ(*f*) and the effective core potential (ECP, LANL2DZ) for Mn atom; 6-31G (*d*') for all other atoms (C, O, and H). BS2: Ahlrichs redefined Def2-TZVP for H, C, O and Mn. BS4: modified-LANL2DZ(*f*) and the effective core potential (ECP, LANL2DZ) for Mn atom; 6-31G (*d*,p) for all other atoms (C, O, and H).

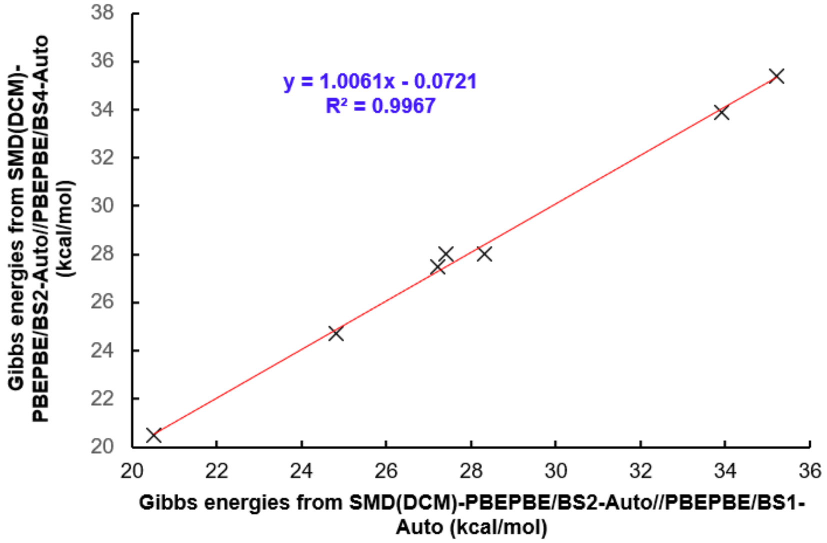

Figure S2. Linear fitting between the Gibbs energies from SMD(DCM)-PBE/BS2-Auto//PBE/BS1-Auto and Gibbs energies from SMD(DCM)-PBE/BS2-Auto//PBE/BS4-Auto.

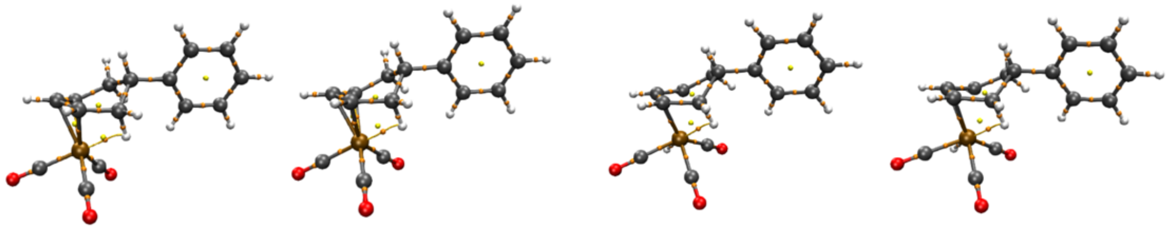

**1**	**1**	**3**	**3**
PBEPBE/BS1-Auto	PBEPBE/BS4-Auto	PBEPBE/BS1-Auto	PBEPBE/BS4-Auto
$\rho_{(BCP, Mn\text{-}H)} = 0.0480$	$\rho_{(BCP, Mn\text{-}H)} = 0.0505$	$\rho_{(BCP, Mn\text{-}H)} = 0.0544$	$\rho_{(BCP, Mn\text{-}H)} = 0.0582$
$\nabla^2\rho_{(BCP, Mn\text{-}H)} = 0.247$	$\nabla^2\rho_{(BCP, Mn\text{-}H)} = 0.246$	$\nabla^2\rho_{(BCP, Mn\text{-}H)} = 0.213$	$\nabla^2\rho_{(BCP, Mn\text{-}H)} = 0.215$
$\rho_{(BCP, C\text{-}H)} = 0.216$	$\rho_{(BCP, C\text{-}H)} = 0.221$	$\rho_{(BCP, C\text{-}H)} = 0.208$	$\rho_{(BCP, C\text{-}H)} = 0.212$
$\nabla^2\rho_{(BCP, C\text{-}H)} = -0.494$	$\nabla^2\rho_{(BCP, C\text{-}H)} = -0.518$	$\nabla^2\rho_{(BCP, C\text{-}H)} = -0.451$	$\nabla^2\rho_{(BCP, C\text{-}H)} = -0.495$

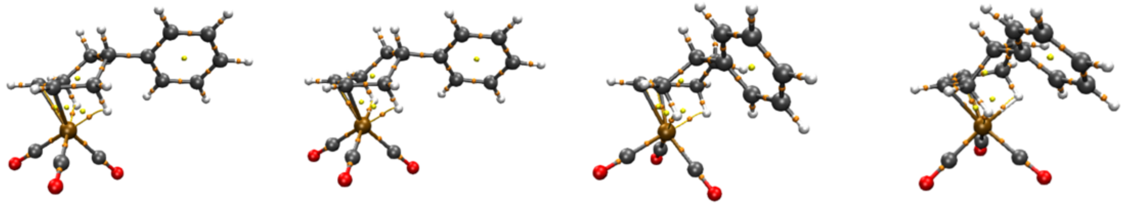

**4**	**4**	**4***	**4***
PBEPBE/BS1-Auto	PBEPBE/BS4-Auto	PBEPBE/BS1-Auto	PBEPBE/BS4-Auto
$\rho_{(BCP, Mn\text{-}H)} = 0.0520$	$\rho_{(BCP, Mn\text{-}H)} = 0.0554$	$\rho_{(BCP, Mn\text{-}H)} = 0.0531$	$\rho_{(BCP, Mn\text{-}H)} = 0.0564$
$\nabla^2\rho_{(BCP, Mn\text{-}H)} = 0.235$	$\nabla^2\rho_{(BCP, Mn\text{-}H)} = 0.237$	$\nabla^2\rho_{(BCP, Mn\text{-}H)} = 0.234$	$\nabla^2\rho_{(BCP, Mn\text{-}H)} = 0.236$
$\rho_{(BCP, C\text{-}H)} = 0.209$	$\rho_{(BCP, C\text{-}H)} = 0.214$	$\rho_{(BCP, C\text{-}H)} = 0.207$	$\rho_{(BCP, C\text{-}H)} = 0.212$
$\nabla^2\rho_{(BCP, C\text{-}H)} = -0.458$	$\nabla^2\rho_{(BCP, C\text{-}H)} = -0.488$	$\nabla^2\rho_{(BCP, C\text{-}H)} = -0.441$	$\nabla^2\rho_{(BCP, C\text{-}H)} = -0.470$

Figure S3. The AIM (Atoms-In-Molecules) analysis of complexes **1**, **3**, and **4**.

The orange balls represent the BCP (bond critical point), the yellow balls represent RCP (ring critical point), the green balls represent CCP (cage critical point), and the bond paths are shown in orange. Atom color codes: C, gray; H, white; O, red; Mn, ochre. The electron densities of bond critical points [$\rho_{(BCP)}$] and Laplacian of electron density ($\nabla^2\rho$) are given in a.u.

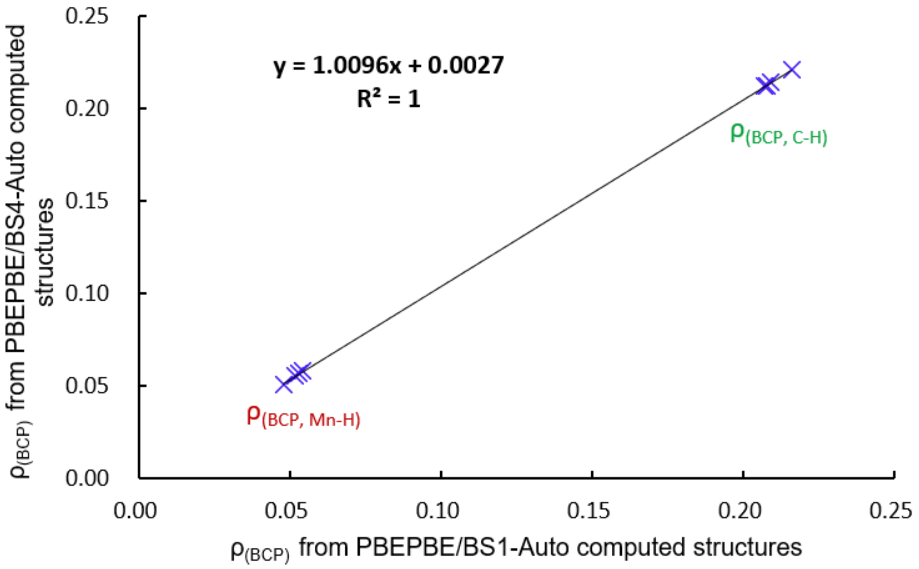

Figure S4. Comparisons of the $\rho_{(BCP)}$ from PBE/BS1-Auto and PBE/BS4-Auto optimized structures**.**

MSD = 0.004, MAD = 0.004. MSD: mean signed deviation. MAD: mean absolute deviation.

BS1: modified-LANL2DZ(*f*) and the effective core potential (ECP, LANL2DZ) for Mn atom; 6-31G (*d'*) for all other atoms (C, O, and H). BS4: modified-LANL2DZ(*f*) and the effective core potential (ECP, LANL2DZ) for Mn atom; 6-31G (*d*,p) for all other atoms (C, O, and H).

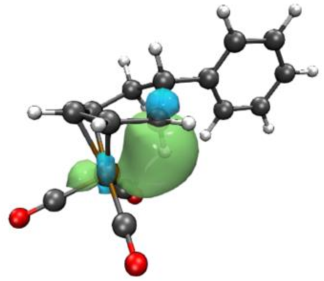

1

Eigenvalue = 0.262

35.5% (H, 1s) + 27.7% (C, 2p)
+ 7.5% (C, 2s) + 17.3% (Mn, 3d)
+ 3.5% (Mn, 3p) + 4.1% (Mn, 3s)

Figure S5. The NAdOs (natural adaptive orbitals, isovalue = 0.05) of the Mn-H-C agostic interaction in complex **1**.

Atom color codes: C, gray; H, white; O, red; Mn, ochre.

Table S6. DFT computed agostic parameters of the agostic complex **1**.

**Mn-H** (Å)	**C-H** (Å)		**Mn-H-C** (°)	$\mathbf{J_{C\text{-}H}}$ (Hz)	
	agostic	non-agostic		agostic	non-agostic
1.854	1.175	1.103	97.4	70.6	126.2
**σ(H)** (ppm)		**Wiberg bond index**			
agostic	non-agostic	Mn-H	C-H agostic	C-H non-agostic	
−8.8	1.8	0.14	0.69	0.87	

Table S7. Donor and acceptor NBOs of the Mn-H-C agostic unit.
The second order interaction energies, $\mathbf{E^{(2)}}$ were given in kcal mol^{-1}.

**Donor → Acceptor**	$\mathbf{E^{(2)}}$ (kcal/mol)										
	**3**	**4**	**4***	**5**	**6**	**7**	**8**	**8***	**9**	**9***	**10**
C-H σ → Mn LP*	63.63	52.70	61.53	49.43	56.39	44.54	43.18	43.58	58.65	28.95	31.98
Mn LP → C-H σ*	1.00	2.42	2.19	1.83	1.68	1.59	1.45	1.37	1.81	0.98	1.24
Mn LP* → C-H σ*	3.12	12.69	11.56	6.13	6.63	25.63	9.46	8.14	14.91	4.12	23.37

The asterisks represent the endo agostic Mn-H-C bond in the di agostic complexes **4**, **8** and **9**.

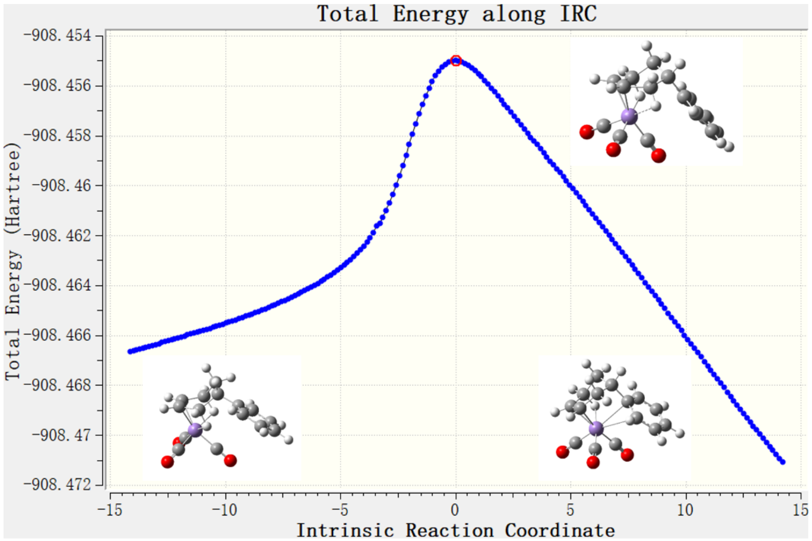

Figure S6. IRC plot for TS-4-6.

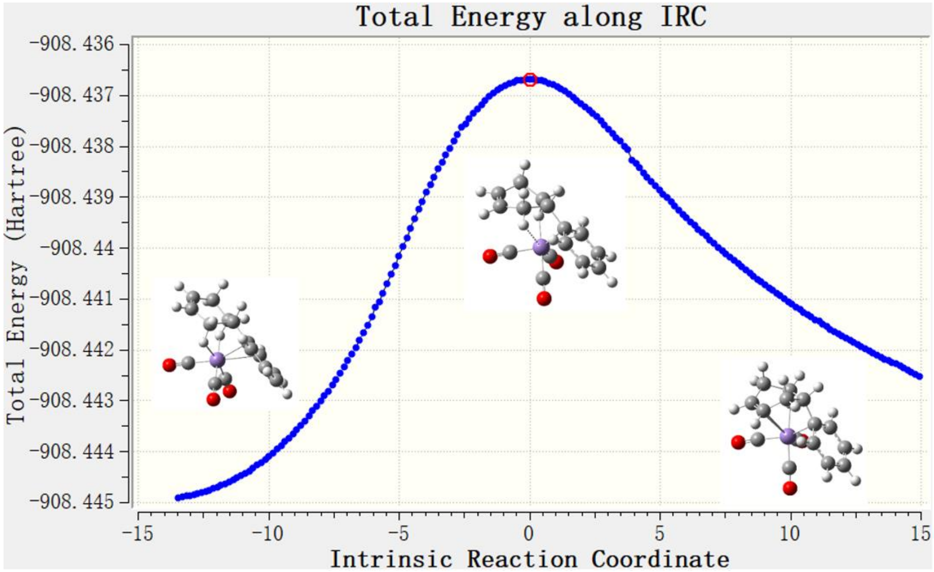

Figure S7. IRC plot for TS-7-8

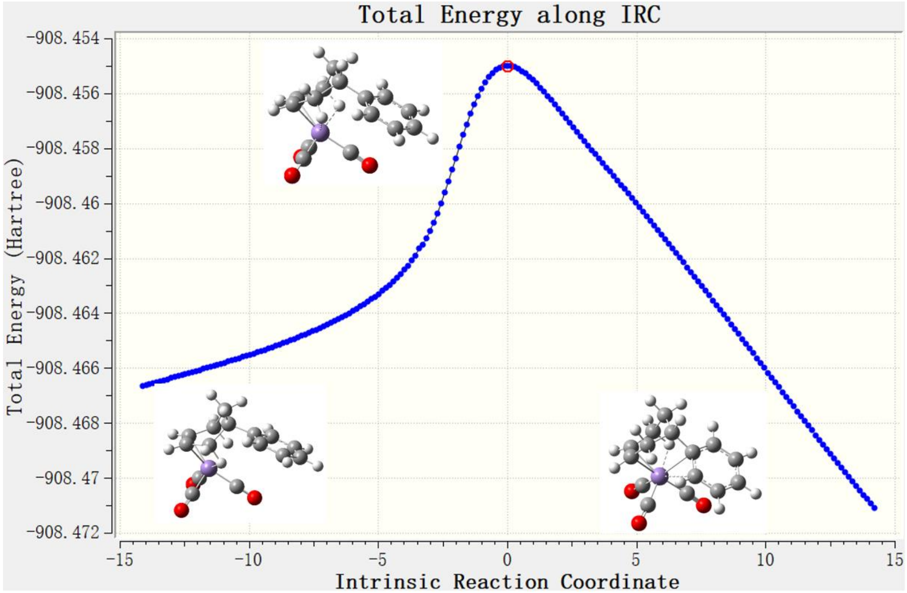

Figure S8. IRC plot for TS-4-13

Table S8. Cartesian coordinates and computed energies (in Hartrees) of optimized structures

32

Complex-1 BS1 el energy= -908.116431686

Mn	-1.964777	3.742769	5.163950
O	-2.180271	6.495689	6.231243
O	0.424282	2.749274	6.586213
O	-3.742647	2.885896	7.328315
C	-3.165083	2.754297	3.761565
C	-2.902840	4.096752	3.374429
C	-1.476789	4.366078	2.983913
C	-0.844998	3.232612	2.120447
C	-0.856714	1.945378	2.975215
C	-2.018173	1.927186	3.966968
C	0.526518	3.612658	1.587944
C	1.628379	3.796873	2.450211
C	2.880073	4.178066	1.945430
C	3.055719	4.377420	0.566977
C	1.969409	4.195552	-0.301720
C	0.717485	3.819581	0.208048
C	-2.081894	5.415094	5.799117
C	-0.536462	3.154067	6.057936
C	-3.026949	3.225741	6.467809
H	-4.171998	2.448738	4.071112
H	-3.691078	4.830812	3.186262
H	-1.346275	5.375828	2.559830
H	-0.770501	4.426152	3.921191
H	-1.522698	3.093896	1.256670
H	-0.931127	1.060937	2.309720
H	0.104115	1.834511	3.511438
H	-2.177425	0.971578	4.482335
H	1.515004	3.638393	3.530717
H	3.722577	4.316196	2.633364
H	4.035356	4.671352	0.172640
H	2.094896	4.345533	-1.380575
H	-0.129104	3.680044	-0.477087

33

Complex-2 BS1 el energy= -908.498490414

Mn	2.191160	1.868482	1.198838
O	3.618943	0.574981	3.435218
O	3.912939	0.419571	-0.711094
O	0.274064	-0.373581	1.141073
C	-3.209844	3.141414	4.023546
C	-2.914798	4.208872	3.257990
C	-1.616105	4.362644	2.500719
C	-0.859824	3.003507	2.410841
C	-0.826765	2.333713	3.800000
C	-2.257282	1.994877	4.255921
C	0.489117	3.244408	1.774936
C	1.650255	3.552927	2.535236
C	2.904999	3.829322	1.904825
C	3.020937	3.786812	0.497214
C	1.871286	3.460002	-0.289395
C	0.637995	3.183110	0.342836
C	3.062105	1.073244	2.552661
C	3.235458	0.980573	0.039548
C	1.035327	0.497586	1.165407
H	-4.187034	3.086702	4.519659
H	-3.640502	5.023820	3.142873
H	-1.813589	4.752249	1.481954
H	-0.967340	5.121282	2.989654
H	-1.441510	2.352076	1.727807
H	-0.201012	1.420922	3.780983
H	-0.376064	3.027192	4.537109
H	-2.623243	1.086101	3.733228
H	-2.245707	1.726863	5.329828
H	1.596458	3.541487	3.628471
H	3.789069	4.025868	2.520454
H	3.989867	3.955257	0.016630
H	1.958460	3.371340	-1.377304
H	-0.221822	2.882376	-0.266666

33

Complex-3 BS1 el energy= -908.431816886

Mn	-2.432522	3.196988	4.561947
O	-5.039718	4.064741	5.637013
O	-1.477515	4.234032	7.168720
O	-1.257795	0.516933	5.116983
C	-3.210428	3.503464	2.591236
C	-2.564347	4.665135	3.137056
C	-1.088760	4.564171	3.264137
C	-0.394419	3.892694	2.027633
C	-0.971224	2.469109	1.899375
C	-2.397001	2.361174	2.416249
C	1.117868	3.968377	2.126778
C	1.839168	3.193059	3.058852
C	3.233086	3.307446	3.154821
C	3.925156	4.198121	2.318039
C	3.217775	4.974485	1.388252
C	1.822525	4.862797	1.297494
C	-4.015154	3.751369	5.202329
C	-1.856941	3.825443	6.157542
C	-1.677911	1.568998	4.894940
H	-4.300799	3.461009	2.485748
H	-3.084430	5.619248	3.269887
H	-0.619009	5.498853	3.612084
H	-0.772562	3.806433	4.118084
H	-0.717539	4.492305	1.155911
H	-1.000301	2.167336	0.832669
H	-0.316472	1.724827	2.387482
H	-2.900066	1.397166	2.262250

H	-3.433301	2.084706	4.949229
H	1.321443	2.480618	3.716274
H	3.780846	2.695454	3.880008
H	5.014897	4.283578	2.390601
H	3.751332	5.668526	0.729677
H	1.274834	5.473249	0.567982

33

Complex-4 BS1 el energy= -908.469060326

Mn	-1.835690	3.792336	5.073703
O	-2.738992	6.486550	5.855535
O	0.688076	4.113288	6.606921
O	-3.248468	2.760520	7.445010
C	-3.123626	2.675492	3.849401
C	-2.962297	3.961312	3.317486
C	-1.593622	4.309838	2.828549
C	-0.933046	3.217403	1.921262
C	-1.070914	1.814224	2.569624
C	-1.904014	1.809448	3.875423
C	0.486616	3.622983	1.555016
C	1.552819	3.482899	2.467940
C	2.843896	3.913705	2.129356
C	3.085843	4.488806	0.871617
C	2.032932	4.631064	-0.044383
C	0.741261	4.203385	0.297701
C	-2.376363	5.433525	5.546160
C	-0.297694	3.994310	6.018572
C	-2.686176	3.158572	6.516578
H	-4.097200	2.302462	4.184972
H	-3.805299	4.651209	3.201618
H	-1.550502	5.316265	2.379212
H	-0.817866	4.457580	3.710778
H	-1.542114	3.238413	0.997782
H	-1.574074	1.124891	1.868459
H	-0.078376	1.379066	2.773787
H	-2.128868	0.794253	4.244474
H	-1.151654	2.148511	4.730863
H	1.390434	3.019650	3.451387
H	3.663878	3.792448	2.845955
H	4.095602	4.819501	0.604991
H	2.215833	5.071541	-1.030650
H	-0.077141	4.313998	-0.425655

33

Complex-5 BS1 el energy= -908.461834176

Mn	0.402100	-0.200233	0.090239
O	-0.291696	2.181071	1.702566
O	0.572984	1.389982	-2.372974
O	3.184481	0.566161	0.629298
C	0.797532	-2.252545	-1.303956
C	1.198263	-2.214525	0.021428
C	0.209831	-2.302647	1.141934
C	-1.266078	-2.554715	0.701948
C	-1.324858	-3.438776	-0.563030
C	-0.470008	-2.933741	-1.745403
C	-1.968867	-1.199638	0.592324
C	-2.007864	-0.425787	-0.604106
C	-2.697799	0.818143	-0.636708
C	-3.341452	1.298950	0.501502
C	-3.302391	0.542106	1.692403
C	-2.617184	-0.675385	1.739529
C	-0.063003	1.245199	1.061787
C	0.505188	0.765115	-1.398031
C	2.091193	0.238558	0.420199
H	1.561403	-2.070846	-2.071888
H	2.264656	-2.178674	0.276766
H	0.567553	-2.974893	1.946718
H	0.252833	-1.296980	1.728820
H	-1.764613	-3.107647	1.518058
H	-0.963623	-4.443162	-0.274245
H	-2.376469	-3.564789	-0.874045
H	-0.130255	-3.801718	-2.348664
H	-1.040442	-2.329114	-2.474308
H	-1.691393	-0.855494	-1.555871
H	-2.740827	1.377743	-1.577987
H	-3.886844	2.248059	0.470676
H	-3.823224	0.903530	2.586199
H	-2.610867	-1.257648	2.669500

33

Complex-6 BS1 el energy= -908.476292385

Mn	-1.291120	3.734241	4.464622
O	-1.666448	6.606715	4.965916
O	1.255894	3.631843	5.981269
O	-2.532054	3.317834	7.094414
C	-3.039572	2.718269	3.811759
C	-3.082114	3.805529	2.941352
C	-2.489059	3.736658	1.552290
C	-1.256141	2.821199	1.506513
C	-1.615338	1.451464	2.125562
C	-2.023517	1.634492	3.597263
C	-0.054928	3.459937	2.217741
C	1.152947	2.718599	2.380034
C	2.335321	3.337035	2.785573
C	2.364493	4.722269	3.067949
C	1.198654	5.472585	2.946239
C	-0.010282	4.853291	2.525745
C	-1.506790	5.476663	4.759845
C	0.288437	3.687911	5.350806
C	-2.055809	3.463564	6.047170
H	-3.806499	2.595655	4.585453
H	-3.836852	4.580325	3.131213
H	-3.281202	3.324453	0.891795
H	-2.280011	4.740072	1.143947
H	-0.967949	2.660583	0.448461
H	-2.462213	1.002726	1.573941

H	-0.779706	0.735370	2.050256
H	-2.318253	0.697901	4.102463
H	-1.041172	1.873443	4.193007
H	1.170295	1.651692	2.129679
H	3.252953	2.744202	2.869951
H	3.302783	5.201054	3.367931
H	1.206448	6.551507	3.137442
H	-0.845232	5.504556	2.250983

33
Complex-7 BS1 el energy= -908.461674257

Mn	-0.243671	0.042830	0.081831
O	0.275569	1.764858	2.395628
O	-2.079355	2.074893	-1.048103
O	1.831089	1.525195	-1.383186
C	1.563119	-1.431152	0.075914
C	0.808473	-1.761566	1.192086
C	-0.157059	-2.919464	1.054956
C	-1.110358	-2.663897	-0.132247
C	-0.304505	-2.101499	-1.360480
C	1.230817	-2.154497	-1.213064
C	-2.178791	-1.635969	0.243931
C	-3.278006	-1.419933	-0.626529
C	-4.281457	-0.501430	-0.306333
C	-4.210604	0.258826	0.882232
C	-3.136153	0.077760	1.749672
C	-2.117327	-0.869943	1.446664
C	0.065616	1.087035	1.477764
C	-1.397583	1.258798	-0.592994
C	1.033058	0.912121	-0.805153
H	2.495508	-0.862904	0.176130
H	1.111552	-1.410106	2.186787
H	0.430146	-3.838593	0.856149
H	-0.723307	-3.114220	1.980518
H	-1.604932	-3.609349	-0.422200
H	-0.641507	-2.568363	-2.303754
H	-0.596214	-1.019629	-1.573208
H	1.591275	-3.202732	-1.172557
H	1.714666	-1.682221	-2.085054
H	-3.355623	-2.008002	-1.549294
H	-5.134557	-0.375628	-0.982478
H	-5.003912	0.973400	1.125609
H	-3.080206	0.635596	2.691322
H	-1.421120	-1.139641	2.247283

33
Complex-8 BS1 el energy= -908.444995568

Mn	-1.592317	0.149089	0.585276
O	-4.392468	0.982429	0.871154
O	-0.952269	2.909651	-0.232977
O	-1.180918	0.869719	3.400352
C	0.607437	-0.174698	-2.682229
C	1.212416	-0.161170	-1.476199
C	0.633438	-0.839764	-0.271170
C	-0.537286	-1.823994	-0.569996
C	-1.468361	-1.192354	-1.634099
C	-0.719227	-0.833724	-2.925098
C	-1.273335	-2.127501	0.748409
C	-2.696774	-2.085045	0.849094
C	-3.349066	-2.407126	2.067352
C	-2.604740	-2.784595	3.182746
C	-1.195989	-2.850797	3.096404
C	-0.542460	-2.524832	1.908716
C	-3.291931	0.637790	0.751095
C	-1.195984	1.822681	0.082400
C	-1.335021	0.584178	2.288293
H	1.092993	0.306683	-3.539833
H	2.175008	0.347173	-1.342473
H	1.419701	-1.358984	0.307917
H	0.342809	-0.036117	0.501637
H	-0.110558	-2.753098	-0.998673
H	-2.322197	-1.856989	-1.853334
H	-1.973611	-0.225022	-1.287095
H	-0.566408	-1.770676	-3.502893
H	-1.353501	-0.200582	-3.574067
H	-3.315542	-1.949022	-0.044235
H	-4.443458	-2.383768	2.109055
H	-3.108753	-3.046435	4.119299
H	-0.609515	-3.167604	3.965772
H	0.549253	-2.605978	1.862940

33
Complex-9 BS1 el energy= -908.444172411

Mn	-1.178974	0.167731	0.755962
O	-3.880790	1.054841	1.506496
O	-0.586499	2.822523	-0.382685
O	-0.230161	1.165327	3.340387
C	0.669629	-0.548319	-2.745168
C	1.308718	-0.685904	-1.565147
C	0.632227	-1.202803	-0.329923
C	-0.725427	-1.914153	-0.586622
C	-1.543069	-1.093431	-1.622440
C	-0.782238	-0.873898	-2.936882
C	-1.534838	-2.083568	0.716681
C	-2.920636	-2.426210	0.686772
C	-3.633833	-2.661677	1.863150
C	-3.000367	-2.557324	3.119878
C	-1.646063	-2.231894	3.186446
C	-0.908901	-2.001576	1.998233
C	-2.821777	0.700327	1.199453
C	-0.822292	1.778282	0.059010
C	-0.600564	0.758733	2.319158
H	1.225192	-0.195461	-3.622753
H	2.369590	-0.425099	-1.471247
H	1.305229	-1.870614	0.242453
H	0.588229	-0.311762	0.417007

H	-0.500486	-2.907393	-1.025887
H	-2.515597	-1.572681	-1.819941
H	-1.831765	-0.079062	-1.208765
H	-0.867683	-1.798841	-3.547905
H	-1.276482	-0.089889	-3.541900
H	-3.435602	-2.539152	-0.272723
H	-4.692791	-2.935957	1.805278
H	-3.567708	-2.747307	4.037363
H	-1.131902	-2.180149	4.152296
H	0.178420	-1.898325	2.082250

33
Complex-10 BS1 el energy= -908.449434921

Mn	0.768184	0.562103	0.004116
O	3.612654	0.497028	0.726551
O	0.329458	2.258318	2.360845
O	1.407746	2.890347	-1.674527
C	-4.157335	0.521367	-0.152003
C	-3.133983	1.133075	-0.788425
C	-1.782596	0.497488	-0.932283
C	-1.715345	-0.976580	-0.470235
C	-2.555083	-1.175152	0.805426
C	-4.025192	-0.797115	0.558613
C	-0.244640	-1.374811	-0.334646
C	0.347478	-1.746518	0.903916
C	1.644089	-2.336486	0.943474
C	2.408604	-2.464767	-0.219832
C	1.906599	-1.928764	-1.423175
C	0.602687	-1.380544	-1.486334
C	2.490514	0.483239	0.436329
C	0.505334	1.602145	1.421141
C	1.149288	1.982459	-1.002280
H	-5.146322	0.997618	-0.158245
H	-3.274860	2.109208	-1.267956
H	-1.410072	0.617883	-1.966807
H	-1.069522	1.142539	-0.285363
H	-2.157450	-1.601503	-1.272614
H	-2.485172	-2.221265	1.155050
H	-2.157304	-0.530481	1.615158
H	-4.539191	-1.586156	-0.029556
H	-4.568801	-0.757011	1.521664
H	-0.250396	-1.727712	1.820570
H	2.030286	-2.700639	1.902587
H	3.394597	-2.938979	-0.192469
H	2.510871	-1.969742	-2.337153
H	0.196518	-1.086197	-2.461990

33
Complex-11 BS1 el energy= -908.495538845

Mn	1.496259	-0.026448	0.091167
O	4.087486	0.691724	1.304951
O	0.166143	1.747101	2.037466
O	1.778685	2.237633	-1.782411
C	-4.489299	0.373352	-0.199746
C	-3.561967	1.031016	-0.920195
C	-2.107505	0.628695	-0.971024
C	-1.885412	-0.805199	-0.418012
C	-2.662156	-0.994219	0.900228
C	-4.175002	-0.816225	0.670056
C	-0.414624	-1.190463	-0.352553
C	0.221610	-1.650096	0.853211
C	1.571419	-2.080643	0.873400
C	2.355765	-2.017859	-0.317991
C	1.772751	-1.526989	-1.509095
C	0.407268	-1.106125	-1.512253
C	3.069011	0.417536	0.830745
C	0.687483	1.060227	1.265246
C	1.671658	1.356290	-1.041341
H	-5.534394	0.706232	-0.232088
H	-3.845876	1.904016	-1.520910
H	-1.742964	0.700454	-2.015219
H	-1.491544	1.348546	-0.390410
H	-2.324638	-1.502744	-1.164646
H	-2.476844	-1.996621	1.330840
H	-2.315539	-0.245923	1.641442
H	-4.603231	-1.735569	0.217989
H	-4.682419	-0.713117	1.648291
H	-0.346713	-1.657263	1.788091
H	2.024590	-2.409087	1.814170
H	3.412304	-2.304819	-0.298917
H	2.376498	-1.424341	-2.416482
H	-0.015160	-0.696675	-2.436456

33
Complex-12 BS1 el energy= -908.495678245

Mn	-1.359334	-0.026451	0.124154
O	-0.120899	1.379634	2.401996
O	-3.985947	0.307902	1.425865
O	-1.736017	2.556690	-1.253856
C	4.647330	0.539719	0.132010
C	4.201735	-0.444752	0.934899
C	2.784618	-0.970974	0.900142
C	2.064007	-0.558464	-0.399297
C	2.281301	0.955880	-0.659430
C	3.775477	1.261606	-0.862764
C	0.609433	-0.994834	-0.477609
C	-0.176503	-0.694317	-1.626478
C	-1.521611	-1.154526	-1.770017
C	-2.122995	-1.908728	-0.736577
C	-1.372612	-2.204746	0.442159
C	-0.042251	-1.736495	0.569583
C	-0.607100	0.836315	1.502894
C	-2.954090	0.183339	0.918942
C	-1.593987	1.548426	-0.705443
H	5.697113	0.853151	0.194042
H	4.879701	-0.917593	1.655757

H	2.798857	-2.075685	0.995470
H	2.230024	-0.593606	1.787690
H	2.560496	-1.100724	-1.234388
H	1.705546	1.306105	-1.536089
H	1.902766	1.516019	0.217953
H	4.086376	0.999154	-1.896011
H	3.936693	2.354072	-0.781457
H	0.256619	-0.080726	-2.423972
H	-2.098966	-0.878953	-2.658455
H	-3.167073	-2.227267	-0.821518
H	-1.840425	-2.747177	1.270001
H	0.496824	-1.933105	1.500965

33
Complex-13 BS1 el energy= -908.495969701

Mn	-2.629959	0.067721	0.853760
O	-4.888860	1.663731	1.876365
O	-3.671837	0.480663	-1.874636
O	-1.032299	2.538387	0.625430
C	2.028060	-1.140930	-0.694517
C	1.799393	-2.421218	-0.333028
C	0.515699	-3.162126	-0.617015
C	-0.672307	-2.225221	-0.997115
C	-0.169063	-1.114044	-1.959992
C	1.017903	-0.294969	-1.426314
C	-1.376694	-1.757465	0.264409
C	-2.757614	-2.099466	0.487621
C	-3.425930	-1.799466	1.698986
C	-2.737406	-1.084512	2.726567
C	-1.394912	-0.692273	2.519376
C	-0.730421	-1.014556	1.294612
C	-4.003214	1.041458	1.468591
C	-3.254185	0.324697	-0.806264
C	-1.665933	1.573953	0.711340
H	2.995064	-0.677201	-0.459883
H	2.585571	-2.989578	0.180614
H	0.671085	-3.865524	-1.460860
H	0.242948	-3.805626	0.243058
H	-1.414213	-2.827442	-1.554318
H	0.147844	-1.638234	-2.880696
H	-1.003150	-0.452333	-2.254636
H	1.510266	0.215837	-2.276841
H	0.673671	0.538115	-0.775088
H	-3.311249	-2.607301	-0.310761
H	-4.480077	-2.066739	1.823686
H	-3.257800	-0.806318	3.648909
H	-0.872840	-0.100383	3.278145
H	0.307823	-0.699830	1.149717

33
TS-4-6 BS1 el energy= -908.454931207

Mn	0.084566	-0.067363	-0.053198
O	1.362992	0.937628	2.396447
O	-0.478877	2.712764	-0.956984
O	2.727707	0.047519	-1.299919
C	0.139745	-2.155820	-0.298099
C	-0.364799	-1.906534	0.974249
C	-1.765786	-1.370908	1.209705
C	-2.755535	-1.332269	-0.012097
C	-2.208042	-2.184209	-1.169373
C	-0.737132	-1.809247	-1.463280
C	-3.105207	0.112265	-0.417964
C	-3.179704	0.539648	-1.765048
C	-3.574479	1.846094	-2.092805
C	-3.919607	2.756476	-1.083753
C	-3.876054	2.347055	0.259256
C	-3.473334	1.046131	0.584922
C	0.846211	0.549759	1.435360
C	-0.280283	1.631187	-0.607619
C	1.676926	0.005398	-0.812547
H	1.096026	-2.671058	-0.441270
H	0.205117	-2.216704	1.858551
H	-2.181371	-1.946156	2.062610
H	-1.686291	-0.343211	1.645755
H	-3.695459	-1.804337	0.335326
H	-2.232052	-3.254341	-0.896451
H	-2.830154	-2.079370	-2.072252
H	-0.335166	-2.205026	-2.410940
H	-0.726856	-0.659370	-1.684160
H	-2.940077	-0.144430	-2.586490
H	-3.616307	2.147935	-3.145117
H	-4.231427	3.774487	-1.340998
H	-4.166165	3.039740	1.056877
H	-3.487738	0.744226	1.640265

33
TS-6-7 BS1 el energy= -908.460709509

Mn	2.681584	-5.875484	-2.713103
O	2.894250	-3.909874	-0.562716
O	0.881837	-4.041552	-4.184418
O	4.771848	-4.418176	-4.184932
C	4.622612	-7.146124	-2.386554
C	3.793141	-7.454186	-1.318417
C	2.998940	-8.738069	-1.411445
C	2.196128	-8.736444	-2.726626
C	3.137187	-8.433000	-3.957437
C	4.596724	-8.076835	-3.577385
C	1.064733	-7.702821	-2.645260
C	0.143683	-7.609546	-3.725974
C	-0.995383	-6.800006	-3.646032
C	-1.231587	-6.007422	-2.503835
C	-0.322520	-6.041071	-1.445241
C	0.817637	-6.891028	-1.497071
C	2.814105	-4.685104	-1.423520
C	1.537271	-4.794021	-3.598520
C	3.970399	-5.013693	-3.593263

H	5.460013	-6.450397	-2.252567
H	3.936579	-6.962054	-0.347777
H	3.707676	-9.591144	-1.412902
H	2.339494	-8.893546	-0.541630
H	1.728847	-9.728485	-2.872086
H	3.137345	-9.281069	-4.664205
H	2.717794	-7.587989	-4.552684
H	5.156447	-8.996096	-3.305230
H	5.119279	-7.642863	-4.447048
H	0.304637	-8.225903	-4.619223
H	-1.706917	-6.785467	-4.479220
H	-2.122748	-5.373508	-2.447899
H	-0.497263	-5.441310	-0.544760
H	1.381545	-7.039680	-0.571010

33

TS-7-8 BS1 el energy= -908.436724935

Mn	0.982668	-0.229078	5.379506
O	3.919443	0.074028	5.417787
O	0.802185	2.661510	5.854112
O	1.002968	0.340653	2.512405
C	2.939634	-3.222381	6.613382
C	2.565338	-2.419977	7.633222
C	1.118133	-2.191816	8.000277
C	0.091051	-2.338578	6.819194
C	0.741821	-2.851971	5.502369
C	1.899315	-3.838509	5.719072
C	-0.732843	-1.056274	6.537737
C	-1.052403	-0.120041	7.560622
C	-1.918146	0.962074	7.322120
C	-2.465165	1.175287	6.046314
C	-2.151641	0.286852	5.010203
C	-1.300759	-0.823741	5.241576
C	2.771221	-0.076589	5.419423
C	0.859930	1.516927	5.679822
C	1.010830	0.111539	3.650074
H	3.998907	-3.427542	6.419625
H	3.319059	-1.972470	8.293221
H	0.843047	-2.916074	8.793135
H	1.030918	-1.204763	8.485774
H	-0.644490	-3.113908	7.115871
H	-0.030393	-3.282775	4.840356
H	1.208280	-2.036348	4.846704
H	1.485175	-4.763383	6.175522
H	2.323502	-4.142938	4.746396
H	-0.664543	-0.263476	8.574010
H	-2.161965	1.642489	8.145505
H	-3.135154	2.022066	5.864661
H	-2.591273	0.420472	4.015081
H	-1.218451	-1.577412	4.450857

33

TS-8-9 BS1 el energy= -908.443624106

Mn	-0.043974	-0.019279	0.090284
O	1.521366	0.836754	2.425946
O	-1.912043	2.213352	0.539580
O	1.621116	1.768843	-1.529899
C	-3.647542	-1.618691	-0.246389
C	-2.952813	-1.250928	-1.342370
C	-1.464530	-1.406349	-1.453198
C	-0.820072	-2.322256	-0.372361
C	-1.472163	-2.015374	1.002685
C	-2.997422	-2.186407	0.980899
C	0.713910	-2.120298	-0.329796
C	1.474369	-2.383657	0.849759
C	2.874984	-2.333498	0.839704
C	3.564034	-2.019374	-0.343105
C	2.847068	-1.740875	-1.515801
C	1.443982	-1.789526	-1.512516
C	0.903055	0.481114	1.512435
C	-1.178025	1.334398	0.364616
C	0.964863	1.048009	-0.901903
H	-4.739275	-1.513928	-0.234982
H	-3.468078	-0.828813	-2.213475
H	-1.183496	-1.763474	-2.462166
H	-1.004515	-0.343318	-1.507140
H	-1.055213	-3.373410	-0.637110
H	-1.032609	-2.642065	1.795751
H	-1.265664	-0.953938	1.361500
H	-3.222418	-3.273027	1.045737
H	-3.445759	-1.749378	1.893369
H	0.969898	-2.665768	1.779150
H	3.427958	-2.548761	1.760251
H	4.659176	-1.990977	-0.349135
H	3.375123	-1.494387	-2.443064
H	0.914311	-1.614553	-2.454947

33

TS-9-10 BS1 el energy= -908.443611054

Mn	0.306019	0.626960	0.163028
O	2.532429	0.226057	2.041684
O	-0.532909	3.008519	1.687027
O	2.063528	2.315965	-1.450157
C	-3.999915	0.621240	-0.022975
C	-3.146372	0.969969	-1.008786
C	-1.839315	0.272390	-1.238618
C	-1.651229	-1.041914	-0.432986
C	-2.227294	-0.873168	0.997501
C	-3.705225	-0.457034	0.978171
C	-0.169525	-1.478194	-0.426227
C	0.347071	-2.355387	0.571709
C	1.660443	-2.841302	0.507312
C	2.521651	-2.444689	-0.532751
C	2.055134	-1.566204	-1.514556
C	0.721644	-1.083024	-1.474480
C	1.647507	0.365675	1.307167

C	-0.198126	2.073912	1.090445
C	1.359439	1.652997	-0.809007
H	-4.968355	1.131368	0.053290
H	-3.401972	1.772883	-1.710629
H	-1.685802	0.084467	-2.319004
H	-1.016989	1.076437	-1.063681
H	-2.245312	-1.823834	-0.950011
H	-2.117358	-1.805543	1.576057
H	-1.639289	-0.104054	1.556877
H	-4.335918	-1.344169	0.752535
H	-4.020527	-0.134937	1.988892
H	-0.297037	-2.688824	1.391306
H	2.014456	-3.533505	1.279151
H	3.546927	-2.826998	-0.576377
H	2.704054	-1.268135	-2.345788
H	0.349808	-0.533527	-2.346570

33

TS-10-11 BS1 el energy= -908.449267848

Mn	0.815858	0.507129	-0.013636
O	3.595652	0.558389	0.919002
O	0.128920	2.217496	2.271096
O	1.467222	2.836818	-1.683244
C	-4.196608	0.582971	-0.095289
C	-3.193810	1.128616	-0.819940
C	-1.870793	0.453044	-1.004123
C	-1.798194	-0.990472	-0.446559
C	-2.587227	-1.101896	0.871929
C	-4.058524	-0.701022	0.673807
C	-0.328412	-1.394587	-0.344552
C	0.286741	-1.782187	0.875443
C	1.604383	-2.330694	0.892544
C	2.374150	-2.372742	-0.271440
C	1.847000	-1.796249	-1.450638
C	0.508881	-1.337228	-1.502030
C	2.498421	0.509168	0.547305
C	0.404616	1.551510	1.362499
C	1.206539	1.924510	-1.017576
H	-5.171412	1.086929	-0.071084
H	-3.338994	2.081429	-1.343465
H	-1.565337	0.490677	-2.066915
H	-1.079243	1.101702	-0.471481
H	-2.275464	-1.662856	-1.188767
H	-2.528794	-2.130880	1.271464
H	-2.138979	-0.428941	1.630698
H	-4.617283	-1.505686	0.151051
H	-4.554231	-0.601511	1.658216
H	-0.300373	-1.794219	1.798730
H	2.004749	-2.716052	1.837379
H	3.381861	-2.799710	-0.265775
H	2.457186	-1.753378	-2.360639
H	0.091620	-1.026521	-2.467079

33

TS-11-12 BS1 el energy= -908.494437443

Mn	-0.009967	-0.042990	0.096492
O	-2.773805	0.986137	0.007339
O	0.914251	2.701757	-0.467025
O	0.092272	0.549079	2.986232
C	6.018494	0.445395	0.042338
C	5.381671	-0.137289	1.074416
C	3.957850	-0.638296	1.007752
C	3.416933	-0.689353	-0.440909
C	3.826023	0.589724	-1.207891
C	5.358700	0.716288	-1.283918
C	1.939029	-1.054067	-0.521920
C	1.125161	-0.725537	-1.662951
C	-0.215097	-1.165533	-1.788928
C	-0.817715	-1.921731	-0.738909
C	-0.062142	-2.227004	0.416059
C	1.290379	-1.777403	0.521266
C	-1.688268	0.589414	0.044659
C	0.563484	1.622083	-0.242752
C	0.046495	0.325393	1.852430
H	7.065896	0.752007	0.155479
H	5.901602	-0.290871	2.028108
H	3.923284	-1.646727	1.467330
H	3.299467	0.004410	1.633764
H	3.924653	-1.542849	-0.946698
H	3.421131	0.602025	-2.236082
H	3.412924	1.471974	-0.682238
H	5.762769	0.028517	-2.056611
H	5.619703	1.732647	-1.636079
H	1.543173	-0.103645	-2.460008
H	-0.802146	-0.876651	-2.666530
H	-1.868319	-2.222715	-0.807795
H	-0.524264	-2.759581	1.253394
H	1.836202	-1.986375	1.445832

33

TS-2-12 BS1 el energy= -908.494681203

Mn	0.757804	-2.388289	-1.644794
O	2.269755	-3.019294	0.808073
O	-1.659145	-2.083549	0.017323
O	1.340340	0.478792	-1.262504
C	6.664573	-3.053272	-3.051252
C	6.043315	-4.197163	-3.395349
C	4.544368	-4.381712	-3.325311
C	3.823754	-3.015403	-3.274304
C	4.473104	-2.124763	-2.178951
C	5.944997	-1.837914	-2.524667
C	2.313176	-3.129282	-3.162582
C	1.475649	-2.120098	-3.725001
C	0.053186	-2.221053	-3.731291
C	-0.569373	-3.339633	-3.122535
C	0.231133	-4.345264	-2.507551

C	1.645194	-4.215269	-2.504203
C	1.681382	-2.761969	-0.154663
C	-0.706968	-2.199811	-0.628590
C	1.110467	-0.646456	-1.402969
H	7.754895	-2.979563	-3.151109
H	6.625088	-5.052805	-3.759375
H	4.191020	-4.964668	-4.198799
H	4.296367	-4.993454	-2.431247
H	4.013682	-2.511200	-4.244296
H	3.918102	-1.174601	-2.067486
H	4.410989	-2.656296	-1.209478
H	6.013253	-1.013263	-3.265179
H	6.460876	-1.457342	-1.621515
H	1.946070	-1.234981	-4.170228
H	-0.551728	-1.418121	-4.163890
H	-1.661776	-3.410518	-3.089745
H	-0.240993	-5.186735	-1.990954
H	2.240044	-4.975451	-1.987801

33

TS-3-4 BS1 el energy= -908.430964240

Mn	0.063121	0.018894	0.090901
O	-0.467719	2.040582	-2.025698
O	0.731258	2.134860	2.065979
O	-2.826015	-0.469919	-0.273473
C	0.295793	-2.085124	0.247026
C	0.739887	-1.559504	-1.009762
C	1.988515	-0.749961	-0.936582
C	3.083854	-1.343988	0.014510
C	2.469415	-1.394477	1.425453
C	0.962632	-1.595709	1.400085
C	4.392559	-0.584163	-0.097990
C	4.543506	0.717022	0.425968
C	5.752224	1.411119	0.275039
C	6.827840	0.812447	-0.400967
C	6.688501	-0.480594	-0.926831
C	5.476783	-1.171291	-0.779675
C	-0.253005	1.255721	-1.208196
C	0.467763	1.323533	1.285989
C	-1.694354	-0.283836	-0.134028
H	-0.613717	-2.694053	0.315040
H	0.318658	-1.880175	-1.967602
H	2.350265	-0.425687	-1.926829
H	1.803119	0.294431	-0.410014
H	3.247220	-2.377489	-0.345255
H	2.897384	-2.243974	1.995666
H	2.736306	-0.495005	2.009979
H	0.485030	-1.773141	2.372757
H	-0.768422	0.143670	1.387986
H	3.720985	1.202687	0.969452
H	5.856563	2.419091	0.691692
H	7.773359	1.353934	-0.514636
H	7.524528	-0.955566	-1.451796
H	5.374337	-2.182827	-1.193331

33

TS-4-5 BS1 el energy= -908.454311553

Mn	0.510222	-0.044697	-0.149315
O	-1.574760	2.076243	-0.133197
O	1.625785	1.018479	-2.662292
O	2.324248	1.747212	1.274908
C	1.654843	-1.895016	-0.626648
C	1.495076	-1.678292	0.740675
C	0.130628	-1.705131	1.348277
C	-0.978673	-2.545092	0.613675
C	-0.359615	-3.403222	-0.507554
C	0.599309	-2.627981	-1.430934
C	-2.135665	-1.628412	0.180453
C	-2.513881	-1.415649	-1.166132
C	-3.598824	-0.577496	-1.486945
C	-4.325629	0.056359	-0.472749
C	-3.972598	-0.158694	0.873032
C	-2.890097	-0.985481	1.192288
C	-0.784779	1.235344	-0.145647
C	1.179487	0.599200	-1.680150
C	1.602992	1.036806	0.709333
H	2.657371	-1.782790	-1.057193
H	2.352851	-1.432575	1.378855
H	0.184533	-1.879535	2.438658
H	-0.270975	-0.587502	1.415967
H	-1.387155	-3.237984	1.373479
H	0.212098	-4.225137	-0.039043
H	-1.163091	-3.875974	-1.099006
H	1.119344	-3.314712	-2.126643
H	0.049562	-1.934427	-2.104084
H	-2.004493	-1.938171	-1.981716
H	-3.877444	-0.436592	-2.537085
H	-5.174189	0.702359	-0.722344
H	-4.554029	0.309083	1.675229
H	-2.645640	-1.164121	2.248188

33

TS-5-8 BS1 el energy= -908.435812545

Mn	-0.257125	0.786701	-0.049952
O	2.071593	2.135203	-1.203995
O	0.310367	2.020906	2.560539
O	-1.772970	3.214204	-0.715010
C	-3.314708	-0.796975	0.220400
C	-2.568932	-0.208191	-0.758411
C	-1.323196	-0.841303	-1.306301
C	-0.628331	-1.915452	-0.380794
C	-1.358577	-2.130529	0.967693
C	-2.887063	-2.066835	0.882241
C	0.832635	-1.469270	-0.206960
C	1.383938	-1.027125	1.028890
C	2.750518	-0.658564	1.118654

C	3.576914	-0.739375	-0.000299
C	3.048009	-1.191022	-1.230601
C	1.702126	-1.540484	-1.333171
C	1.164517	1.582840	-0.738539
C	0.090872	1.529777	1.533542
C	-1.203122	2.236555	-0.460644
H	-4.284279	-0.356400	0.486071
H	-2.984944	0.640268	-1.315397
H	-1.528284	-1.264576	-2.311792
H	-0.572844	-0.033317	-1.683798
H	-0.630958	-2.874988	-0.931314
H	-1.022085	-3.085628	1.407400
H	-1.078435	-1.335198	1.686170
H	-3.291555	-2.923719	0.300135
H	-3.331175	-2.158146	1.889348
H	0.813129	-1.099339	1.959238
H	3.154030	-0.340359	2.086189
H	4.636609	-0.472947	0.075230
H	3.699857	-1.279110	-2.106730
H	1.309826	-1.907947	-2.290190

33

TS-6-13 BS1 el energy= -908.444314347

Mn	0.242964	-0.741882	0.252738
O	-0.480560	-3.250196	1.581342
O	1.945759	-0.306705	2.609960
O	2.365709	-2.257897	-1.087560
C	1.141250	2.437863	0.721396
C	1.325693	1.798533	-0.628545
C	0.233797	2.171084	-1.629450
C	-1.148320	2.035219	-0.960057
C	-1.234821	3.059965	0.191335
C	-0.020732	3.027218	1.084480
C	-1.487222	0.584612	-0.571811
C	-2.164800	0.243386	0.631753
C	-2.892406	-0.974017	0.750141
C	-2.825899	-1.942095	-0.255022
C	-1.953916	-1.721187	-1.343919
C	-1.311249	-0.469652	-1.521931
C	-0.234501	-2.251709	1.044759
C	1.277762	-0.461432	1.675541
C	1.538302	-1.648894	-0.549562
H	2.002478	2.452015	1.400533
H	2.338612	1.996359	-1.030349
H	0.373814	3.224437	-1.936128
H	0.319608	1.570746	-2.552298
H	-1.927101	2.294836	-1.706524
H	-1.325837	4.071384	-0.256381
H	-2.162351	2.944541	0.782158
H	-0.088952	3.536289	2.054477
H	1.378304	0.650616	-0.541469
H	-2.256622	0.985405	1.430684
H	-3.502271	-1.142181	1.645175
H	-3.392243	-2.875479	-0.178184
H	-1.816759	-2.498604	-2.104905
H	-0.789038	-0.278464	-2.466111

33

TS-2-13 BS1 el energy= -908.488684280

Mn	-1.757884	-2.747984	-1.384489
O	1.060137	-3.064909	-0.566255
O	-2.265381	-1.169040	1.056266
O	-1.033983	-0.274376	-2.824810
C	-7.856721	-2.111853	-1.789252
C	-7.687353	-3.262177	-1.112970
C	-6.322158	-3.848490	-0.844140
C	-5.169428	-2.776189	-0.681784
C	-5.567776	-1.348287	-1.142898
C	-6.666453	-1.294790	-2.225675
C	-3.941660	-3.353771	-1.356337
C	-3.127651	-4.313500	-0.652794
C	-2.010438	-4.929625	-1.260722
C	-1.641288	-4.584323	-2.598976
C	-2.406197	-3.622456	-3.296562
C	-3.539710	-3.007634	-2.675270
C	-0.044350	-2.934021	-0.882779
C	-2.057667	-1.780424	0.096240
C	-1.310009	-1.241293	-2.253820
H	-8.864224	-1.740027	-2.009801
H	-8.548750	-3.855663	-0.782944
H	-6.341969	-4.493899	0.053018
H	-6.071358	-4.531030	-1.684953
H	-4.934227	-2.720796	0.397283
H	-5.960357	-0.819952	-0.256495
H	-4.668844	-0.783630	-1.459535
H	-6.951822	-0.241482	-2.397011
H	-6.288714	-1.662588	-3.204906
H	-3.366785	-4.547987	0.390625
H	-1.395200	-5.629961	-0.686086
H	-0.749535	-5.022182	-3.058241
H	-2.103368	-3.307298	-4.300733
H	-4.088782	-2.233250	-3.219554

32

Complex-1 D3(BJ)-BS1 el energy= -908.161616654

Mn	-1.936269	3.759895	5.128811
O	-2.135511	6.525649	6.160103
O	0.519952	2.781784	6.433452
O	-3.634299	2.914354	7.359400
C	-3.174899	2.746597	3.784037
C	-2.923929	4.080401	3.363277
C	-1.507381	4.345380	2.945455
C	-0.873081	3.205106	2.098023
C	-0.887649	1.922410	2.957860
C	-2.021150	1.926543	3.978090
C	0.504492	3.592853	1.594990

C	1.587610	3.756667	2.483550
C	2.845925	4.157511	2.013230
C	3.045281	4.397092	0.644678
C	1.976435	4.235851	-0.249024
C	0.717369	3.840234	0.226295
C	-2.043890	5.439158	5.742143
C	-0.471297	3.183636	5.962011
C	-2.949774	3.250833	6.473088
H	-4.172735	2.447187	4.125995
H	-3.715540	4.812371	3.183662
H	-1.383414	5.347711	2.503376
H	-0.781957	4.426812	3.869576
H	-1.537084	3.063649	1.224739
H	-0.993245	1.037965	2.297227
H	0.083263	1.796677	3.470713
H	-2.164838	0.983574	4.519917
H	1.454223	3.566522	3.556264
H	3.674883	4.279546	2.719978
H	4.030291	4.706486	0.277064
H	2.121457	4.417378	-1.320315
H	-0.117232	3.717124	-0.476037

33

Complex-2 D3(BJ)-BS1 el energy= -908.544272346

Mn	2.176254	1.880773	1.209704
O	3.662078	0.586105	3.403820
O	3.790258	0.384707	-0.754164
O	0.199494	-0.305473	1.264316
C	-3.205485	3.108268	3.993646
C	-2.921405	4.181969	3.233156
C	-1.618278	4.356835	2.490203
C	-0.846961	3.009200	2.406443
C	-0.808154	2.346352	3.796665
C	-2.234373	1.980519	4.238641
C	0.498746	3.261819	1.777323
C	1.656442	3.560385	2.545018
C	2.915793	3.824038	1.920039
C	3.038047	3.782169	0.512942
C	1.887640	3.471523	-0.278645
C	0.647956	3.209936	0.346122
C	3.082850	1.083990	2.535858
C	3.154867	0.963483	0.019069
C	0.989043	0.540039	1.241635
H	-4.186963	3.035033	4.478211
H	-3.660178	4.983540	3.110501
H	-1.809378	4.744813	1.469952
H	-0.982823	5.121082	2.987130
H	-1.418410	2.348127	1.724632
H	-0.162220	1.448003	3.787218
H	-0.379140	3.054018	4.532779
H	-2.576202	1.064510	3.712717
H	-2.229761	1.715364	5.312942
H	1.596785	3.540879	3.637196
H	3.800291	4.004319	2.539555
H	4.011633	3.934067	0.037256
H	1.979930	3.379228	-1.365572
H	-0.212379	2.916379	-0.265310

33

Complex-3 D3(BJ)-BS1 el energy= -908.478753263

Mn	-2.392604	3.204748	4.548489
O	-4.998020	4.001007	5.676809
O	-1.406567	4.279479	7.125496
O	-1.116078	0.564881	5.051538
C	-3.226955	3.499504	2.604175
C	-2.582078	4.669722	3.131168
C	-1.105822	4.582334	3.230022
C	-0.417632	3.908163	1.996759
C	-0.994998	2.485930	1.865341
C	-2.404819	2.366045	2.415584
C	1.091187	3.968357	2.119212
C	1.778335	3.196667	3.078560
C	3.169934	3.297068	3.209109
C	3.892128	4.169559	2.378952
C	3.217405	4.941469	1.422079
C	1.823774	4.844547	1.297529
C	-3.974900	3.713711	5.221894
C	-1.799180	3.854969	6.126236
C	-1.582321	1.602152	4.854160
H	-4.318666	3.443797	2.526505
H	-3.110442	5.616019	3.281869
H	-0.635077	5.519474	3.568632
H	-0.764232	3.830613	4.083472
H	-0.728200	4.511281	1.123495
H	-1.047120	2.196306	0.796691
H	-0.326150	1.740636	2.330882
H	-2.900817	1.396217	2.278791
H	-3.351909	2.063227	4.953028
H	1.235010	2.497729	3.729669
H	3.692472	2.688702	3.955274
H	4.980326	4.244442	2.477842
H	3.775239	5.621100	0.769013
H	1.299495	5.451989	0.549006

33

Complex-4 D3(BJ)-BS1 el energy= -908.516144787

Mn	-1.731175	3.853379	4.948168
O	-2.690846	6.532410	5.700407
O	0.924070	4.364284	6.172031
O	-2.827779	2.869946	7.499354
C	-3.087853	2.628597	3.936735
C	-3.032033	3.878657	3.307849
C	-1.726335	4.253856	2.691291
C	-1.022107	3.153306	1.830713
C	-1.202859	1.753180	2.467882
C	-1.842093	1.802712	3.875239

C	0.419353	3.569376	1.584285
C	1.430764	3.311741	2.532133
C	2.737554	3.777669	2.326291
C	3.048328	4.506572	1.168093
C	2.049654	4.764184	0.216957
C	0.742624	4.300910	0.426707
C	-2.304898	5.484268	5.403165
C	-0.112914	4.170521	5.704968
C	-2.389111	3.249465	6.499650
H	-4.008766	2.243643	4.386400
H	-3.906067	4.535295	3.243678
H	-1.766577	5.228440	2.176784
H	-0.901337	4.525019	3.506574
H	-1.558097	3.174535	0.863703
H	-1.872169	1.135774	1.843498
H	-0.238544	1.222207	2.519777
H	-1.984531	0.808960	4.331273
H	-0.998072	2.227038	4.590052
H	1.214917	2.721950	3.432986
H	3.515332	3.562244	3.067013
H	4.069923	4.865351	1.003602
H	2.288297	5.322390	-0.694694
H	-0.033458	4.501569	-0.323121

33

Complex-6 D3(BJ)-BS1 el energy= -908.528815157

Mn	-1.284952	3.726001	4.458588
O	-1.660784	6.599467	4.938698
O	1.274893	3.630495	5.947632
O	-2.506714	3.306017	7.093311
C	-3.028889	2.711014	3.817591
C	-3.081727	3.801575	2.951584
C	-2.497119	3.739676	1.559185
C	-1.263672	2.828103	1.501544
C	-1.613765	1.456882	2.119363
C	-2.006993	1.636107	3.594219
C	-0.063032	3.467005	2.208723
C	1.139264	2.721478	2.385607
C	2.319037	3.335958	2.803086
C	2.349912	4.721115	3.083379
C	1.188591	5.475763	2.947274
C	-0.016722	4.860849	2.513614
C	-1.501563	5.467498	4.744341
C	0.300250	3.683663	5.328440
C	-2.036666	3.454472	6.043848
H	-3.789859	2.582170	4.595601
H	-3.836450	4.573485	3.150394
H	-3.292367	3.328477	0.902425
H	-2.291496	4.744929	1.155044
H	-0.981531	2.671877	0.441707
H	-2.465717	1.008921	1.575896
H	-0.777485	0.743489	2.033617
H	-2.286970	0.698318	4.104286
H	-1.017269	1.883749	4.176806
H	1.153308	1.653368	2.141990
H	3.232394	2.739559	2.902752
H	3.285049	5.196277	3.397474
H	1.196090	6.553708	3.141915
H	-0.851083	5.512173	2.238650

33

TS-3-4 D3(BJ)-BS1 el energy= -908.477843979

Mn	0.101166	0.022880	0.087511
O	-0.396246	2.033891	-2.043145
O	0.881688	2.114023	2.041522
O	-2.801648	-0.396980	-0.236399
C	0.277139	-2.083573	0.248968
C	0.728600	-1.573994	-1.010956
C	1.990099	-0.788195	-0.944057
C	3.078899	-1.381047	0.009054
C	2.465433	-1.431801	1.419447
C	0.957172	-1.602306	1.397036
C	4.373380	-0.601185	-0.096268
C	4.485953	0.706503	0.418807
C	5.677004	1.430098	0.271055
C	6.771872	0.853356	-0.392452
C	6.669416	-0.446532	-0.908984
C	5.474617	-1.166596	-0.765716
C	-0.195509	1.253219	-1.218399
C	0.567243	1.314437	1.268345
C	-1.664594	-0.236967	-0.111404
H	-0.646457	-2.669474	0.322493
H	0.292714	-1.882496	-1.965630
H	2.356474	-0.471803	-1.934695
H	1.826798	0.265247	-0.423348
H	3.253002	-2.411863	-0.351858
H	2.879776	-2.292187	1.982424
H	2.750086	-0.540855	2.007745
H	0.477183	-1.763430	2.370956
H	-0.706660	0.181327	1.395503
H	3.647370	1.173697	0.953398
H	5.752906	2.443307	0.680408
H	7.704110	1.417384	-0.503767
H	7.520973	-0.903706	-1.424183
H	5.398218	-2.182868	-1.172555

33

TS-4-6 D3(BJ)-BS1 el energy= -908.505222100

Mn	0.037836	-0.066831	-0.040773
O	1.308323	0.943189	2.407631
O	-0.585796	2.702781	-0.927463
O	2.666913	0.109965	-1.303788
C	0.138360	-2.146656	-0.307679
C	-0.365878	-1.917484	0.968289
C	-1.765288	-1.384819	1.207923
C	-2.755364	-1.345120	-0.010187

C	-2.209306	-2.194850	-1.168114
C	-0.746556	-1.800140	-1.465673
C	-3.088791	0.100149	-0.418072
C	-3.173681	0.517433	-1.766717
C	-3.546570	1.827900	-2.098987
C	-3.857975	2.752896	-1.092202
C	-3.803383	2.353702	0.252834
C	-3.421916	1.048035	0.582750
C	0.794473	0.553146	1.445975
C	-0.367231	1.623978	-0.581736
C	1.620960	0.044075	-0.808940
H	1.102093	-2.644428	-0.459071
H	0.209609	-2.226520	1.848766
H	-2.180493	-1.948862	2.067234
H	-1.678109	-0.352475	1.634484
H	-3.698378	-1.810174	0.336546
H	-2.221237	-3.264018	-0.892995
H	-2.837712	-2.094488	-2.066446
H	-0.342102	-2.179464	-2.418428
H	-0.752189	-0.648106	-1.675555
H	-2.955361	-0.177531	-2.584141
H	-3.595046	2.123334	-3.152511
H	-4.149916	3.775532	-1.353113
H	-4.064936	3.058977	1.048876
H	-3.423968	0.754835	1.640240

32
Complex-1 D3(BJ)-BS2 el energy= -1955.35360262

Mn	-1.927209	3.761177	5.127035
O	-2.108695	6.534416	6.161941
O	0.500102	2.770587	6.491686
O	-3.641432	2.944861	7.366774
C	-3.164897	2.740303	3.772875
C	-2.921812	4.066829	3.345204
C	-1.508331	4.336499	2.951810
C	-0.868240	3.209457	2.100379
C	-0.884208	1.925838	2.947461
C	-2.015555	1.923864	3.961630
C	0.503045	3.598327	1.597599
C	1.583398	3.762201	2.478575
C	2.834886	4.160079	2.007633
C	3.031112	4.395961	0.644828
C	1.965839	4.233681	-0.242100
C	0.713336	3.841470	0.234317
C	-2.023790	5.454578	5.743273
C	-0.464785	3.177297	5.987573
C	-2.959019	3.265068	6.481369
H	-4.160049	2.433895	4.099155
H	-3.712413	4.789734	3.157668
H	-1.375850	5.338289	2.524495
H	-0.788841	4.404940	3.884313
H	-1.525496	3.074599	1.227193
H	-0.988879	1.049732	2.284005
H	0.082333	1.799682	3.457673
H	-2.156016	0.982825	4.497222
H	1.453974	3.574099	3.547131
H	3.661844	4.283163	2.709028
H	4.011282	4.702705	0.276566
H	2.109162	4.411630	-1.309305
H	-0.118788	3.717165	-0.463380

33
Complex-2 D3(BJ)-BS2 el energy= -1955.73082828

Mn	2.178060	1.878974	1.208813
O	3.727388	0.561508	3.357016
O	3.753960	0.397737	-0.809359
O	0.220579	-0.335406	1.289563
C	-3.202255	3.123413	3.979191
C	-2.915487	4.177543	3.207039
C	-1.614392	4.338868	2.469840
C	-0.841998	2.997134	2.412433
C	-0.814461	2.357445	3.806313
C	-2.238227	2.002466	4.243566
C	0.501405	3.247953	1.791215
C	1.652874	3.539280	2.559120
C	2.904927	3.813513	1.938278
C	3.024928	3.791336	0.536915
C	1.880559	3.485005	-0.253495
C	0.647881	3.212110	0.365976
C	3.127942	1.066839	2.516386
C	3.139727	0.967827	-0.022614
C	0.991395	0.517792	1.257562
H	-4.182304	3.060633	4.457519
H	-3.649937	4.975149	3.074068
H	-1.803795	4.707333	1.447809
H	-0.986967	5.109539	2.955458
H	-1.403493	2.328722	1.736601
H	-0.175403	1.460690	3.814500
H	-0.389811	3.071375	4.531341
H	-2.579792	1.085351	3.730363
H	-2.243712	1.753829	5.316383
H	1.591605	3.516052	3.646335
H	3.783949	3.997963	2.555925
H	3.991198	3.960780	0.063444
H	1.970812	3.415207	-1.337481
H	-0.211158	2.932042	-0.245022

32
Complex-1 BS4 el energy= -908.135108765

Mn	-1.957531	3.751991	5.154331
O	-2.166073	6.515970	6.199014
O	0.457024	2.772652	6.545007
O	-3.721331	2.892234	7.334041
C	-3.167036	2.747589	3.767395
C	-2.910170	4.087574	3.370103
C	-1.486964	4.357409	2.978302

C	-0.851333	3.225199	2.117304
C	-0.863877	1.938811	2.972378
C	-2.018309	1.924523	3.969827
C	0.521564	3.607281	1.590954
C	1.621236	3.776266	2.457953
C	2.873570	4.162121	1.961417
C	3.051772	4.381719	0.587422
C	1.967709	4.215459	-0.285525
C	0.714872	3.834718	0.215516
C	-2.071231	5.428138	5.775721
C	-0.516463	3.173324	6.029886
C	-3.009823	3.235395	6.467245
H	-4.169987	2.440934	4.080574
H	-3.699940	4.814971	3.176284
H	-1.355345	5.365439	2.557296
H	-0.781025	4.413787	3.918316
H	-1.523487	3.087287	1.251608
H	-0.946070	1.056565	2.308636
H	0.097716	1.824971	3.502019
H	-2.169731	0.975331	4.494472
H	1.505452	3.601489	3.533692
H	3.713765	4.288134	2.651233
H	4.030926	4.679069	0.200096
H	2.095937	4.381248	-1.359703
H	-0.129805	3.707353	-0.471186

33

Complex-2 BS4 el energy= -908.514504369

Mn	2.200098	1.859042	1.197231
O	3.778345	0.550772	3.325412
O	3.781026	0.419508	-0.840883
O	0.257943	-0.365550	1.260194
C	-3.229893	3.139621	3.987861
C	-2.936253	4.191833	3.203577
C	-1.625458	4.347376	2.469507
C	-0.852766	2.995805	2.421791
C	-0.833410	2.360025	3.825761
C	-2.265820	2.014306	4.266586
C	0.499869	3.236939	1.798530
C	1.659707	3.524891	2.567647
C	2.915116	3.806959	1.942566
C	3.033290	3.794668	0.534955
C	1.883676	3.484901	-0.257423
C	0.648724	3.199988	0.365935
C	3.159327	1.055574	2.484398
C	3.157608	0.977209	-0.037296
C	1.031210	0.499718	1.236604
H	-4.214460	3.082014	4.464378
H	-3.670178	4.991492	3.056630
H	-1.808062	4.712800	1.441633
H	-0.996882	5.120993	2.956041
H	-1.416947	2.323961	1.746718
H	-0.198504	1.456344	3.839108
H	-0.404063	3.076682	4.550236
H	-2.610144	1.089402	3.762660
H	-2.270414	1.776611	5.345182
H	1.603826	3.496163	3.658170
H	3.797596	3.989755	2.561170
H	4.000536	3.972270	0.059473
H	1.971725	3.418238	-1.344856
H	-0.210372	2.915692	-0.248496

33

Complex-3 BS4 el energy= -908.452566169

Mn	-2.419158	3.203806	4.561365
O	-5.080450	3.906364	5.628832
O	-1.508061	4.328960	7.152823
O	-1.189381	0.555281	5.140046
C	-3.212320	3.524803	2.598466
C	-2.563193	4.681909	3.149016
C	-1.089191	4.574388	3.266081
C	-0.398163	3.907325	2.026687
C	-0.981584	2.487949	1.889258
C	-2.403459	2.382418	2.411279
C	1.114188	3.970596	2.125968
C	1.825149	3.190964	3.061327
C	3.219006	3.290324	3.158556
C	3.921232	4.169198	2.319346
C	3.224241	4.949018	1.386180
C	1.828878	4.853303	1.294420
C	-4.033315	3.653017	5.198694
C	-1.868947	3.887388	6.144830
C	-1.636939	1.599583	4.913758
H	-4.300839	3.486375	2.493705
H	-3.080478	5.633129	3.292740
H	-0.611847	5.498528	3.623766
H	-0.780206	3.799921	4.111445
H	-0.715665	4.514857	1.160711
H	-1.015304	2.195290	0.822532
H	-0.328257	1.740513	2.369867
H	-2.909304	1.422634	2.254867
H	-3.358095	2.058302	4.933101
H	1.299283	2.487649	3.719031
H	3.758425	2.676654	3.885524
H	5.009774	4.242801	2.392645
H	3.765737	5.632897	0.726734
H	1.288959	5.465698	0.563682

33

Complex-4 BS4 el energy= -908.487751940

Mn	-1.821904	3.797453	5.055278
O	-2.730609	6.493544	5.838936
O	0.735274	4.139259	6.531808
O	-3.178830	2.748318	7.456316
C	-3.124854	2.668010	3.855946
C	-2.978959	3.950311	3.313776

C	-1.618226	4.306140	2.813601
C	-0.947899	3.216940	1.911433
C	-1.089758	1.812219	2.554449
C	-1.898812	1.814030	3.873354
C	0.475689	3.622752	1.561442
C	1.530941	3.474934	2.484710
C	2.825788	3.903667	2.162919
C	3.082530	4.484528	0.911945
C	2.040823	4.634459	-0.014041
C	0.745175	4.209031	0.311014
C	-2.365658	5.437373	5.529963
C	-0.265249	4.011281	5.963535
C	-2.638013	3.155548	6.514881
H	-4.090319	2.290835	4.202716
H	-3.826649	4.631992	3.205168
H	-1.582595	5.309241	2.362278
H	-0.840147	4.463181	3.694776
H	-1.544145	3.240108	0.982059
H	-1.613399	1.132804	1.862238
H	-0.099551	1.368361	2.738388
H	-2.104475	0.804636	4.261301
H	-1.132279	2.178982	4.706716
H	1.356794	3.006050	3.461018
H	3.636170	3.776518	2.886256
H	4.094112	4.813220	0.658466
H	2.235868	5.078766	-0.994010
H	-0.063642	4.324957	-0.419191

33

Complex-5 BS4 el energy= -908.479294435

Mn	0.416128	-0.195844	0.084490
O	-0.292816	2.164165	1.726819
O	0.573297	1.400659	-2.381480
O	3.209886	0.543995	0.624222
C	0.779524	-2.258261	-1.322751
C	1.198004	-2.212771	-0.004265
C	0.224471	-2.281377	1.127485
C	-1.255769	-2.550319	0.713685
C	-1.325280	-3.444113	-0.543758
C	-0.496560	-2.934088	-1.741577
C	-1.971744	-1.202951	0.601562
C	-1.985052	-0.418752	-0.588899
C	-2.676710	0.823232	-0.626019
C	-3.345142	1.292670	0.501262
C	-3.330812	0.525670	1.684838
C	-2.645337	-0.690921	1.737064
C	-0.052123	1.236074	1.072970
C	0.513159	0.775474	-1.402287
C	2.109686	0.228090	0.414191
H	1.532003	-2.082285	-2.100282
H	2.266449	-2.186013	0.234167
H	0.595393	-2.925545	1.945606
H	0.266047	-1.261048	1.694393
H	-1.736460	-3.099506	1.540109
H	-0.947102	-4.440304	-0.256526
H	-2.378214	-3.582091	-0.836232
H	-0.171282	-3.796650	-2.356309
H	-1.082953	-2.326051	-2.452055
H	-1.648734	-0.838009	-1.536884
H	-2.698602	1.391208	-1.560673
H	-3.889024	2.240241	0.467542
H	-3.868552	0.879648	2.569187
H	-2.657524	-1.278568	2.661082

33

Complex-6 BS4 el energy= -908.493864814

Mn	-1.298305	3.736257	4.471885
O	-1.682294	6.614199	4.961418
O	1.261182	3.626467	5.973088
O	-2.544501	3.300837	7.100623
C	-3.043443	2.722453	3.808330
C	-3.087048	3.805932	2.935208
C	-2.485403	3.736808	1.550835
C	-1.255511	2.819125	1.508651
C	-1.612079	1.452752	2.134505
C	-2.018946	1.647060	3.603851
C	-0.053320	3.457657	2.215475
C	1.155497	2.717334	2.365204
C	2.337730	3.334412	2.769031
C	2.365565	4.716651	3.062562
C	1.198937	5.465471	2.953322
C	-0.010316	4.847852	2.533941
C	-1.518611	5.479934	4.762791
C	0.284806	3.684664	5.350224
C	-2.065212	3.456151	6.052319
H	-3.813252	2.599072	4.576037
H	-3.843319	4.577446	3.119909
H	-3.274656	3.329434	0.887480
H	-2.270229	4.738292	1.146689
H	-0.967537	2.654607	0.453189
H	-2.456307	0.998309	1.587875
H	-0.774806	0.741482	2.064146
H	-2.298888	0.716189	4.122270
H	-1.033822	1.905998	4.189343
H	1.173445	1.654251	2.107085
H	3.255465	2.744279	2.845186
H	3.302455	5.193645	3.362531
H	1.205274	6.540363	3.155388
H	-0.846589	5.498256	2.268191

33

TS-3-4 BS4 el energy= -908.452007695

Mn	0.073421	0.023892	0.084186
O	-0.449743	2.054867	-2.030474
O	0.759354	2.107629	2.085742
O	-2.826120	-0.450617	-0.230793

C	0.293506	-2.085578	0.247082
C	0.735119	-1.566163	-1.012531
C	1.983460	-0.760082	-0.944645
C	3.081677	-1.350252	0.003810
C	2.471508	-1.403406	1.416228
C	0.966149	-1.599222	1.395968
C	4.387721	-0.586535	-0.105300
C	4.524525	0.719608	0.407979
C	5.730062	1.418617	0.265108
C	6.816368	0.819816	-0.391360
C	6.691002	-0.477950	-0.906110
C	5.482301	-1.173791	-0.767849
C	-0.237675	1.264073	-1.212947
C	0.488317	1.306860	1.291484
C	-1.687423	-0.269606	-0.111379
H	-0.615469	-2.690883	0.319375
H	0.310273	-1.888630	-1.965409
H	2.342845	-0.434086	-1.932274
H	1.794844	0.282838	-0.411960
H	3.248976	-2.380507	-0.357672
H	2.897905	-2.254298	1.981172
H	2.743491	-0.507449	1.999993
H	0.489893	-1.768764	2.368390
H	-0.748736	0.172707	1.363118
H	3.693923	1.204211	0.936058
H	5.823795	2.429392	0.672012
H	7.758458	1.364490	-0.498530
H	7.534749	-0.951798	-1.415339
H	5.389701	-2.187876	-1.172276

33

TS-4-6 BS4 el energy= -908.472850580

Mn	0.085039	-0.071628	-0.051668
O	1.338242	0.943878	2.412892
O	-0.494150	2.701339	-0.976966
O	2.732381	0.043574	-1.298039
C	0.139759	-2.161146	-0.293224
C	-0.368702	-1.914285	0.976969
C	-1.767231	-1.373229	1.208191
C	-2.752009	-1.331360	-0.016048
C	-2.200822	-2.176626	-1.176127
C	-0.728872	-1.801739	-1.458469
C	-3.101055	0.113822	-0.418466
C	-3.193805	0.535109	-1.765392
C	-3.591856	1.839233	-2.092648
C	-3.921817	2.753470	-1.083140
C	-3.859026	2.350522	0.259946
C	-3.452538	1.051872	0.585467
C	0.832599	0.551300	1.443677
C	-0.289924	1.620222	-0.617433
C	1.678743	0.000863	-0.808421
H	1.091901	-2.680004	-0.433762
H	0.195552	-2.230599	1.860006
H	-2.185490	-1.943238	2.060090
H	-1.684341	-0.345830	1.640220
H	-3.692019	-1.802942	0.325383
H	-2.227924	-3.246626	-0.912355
H	-2.817452	-2.061491	-2.078716
H	-0.322831	-2.185448	-2.406585
H	-0.715659	-0.648307	-1.665959
H	-2.966534	-0.152051	-2.585017
H	-3.647857	2.136894	-3.143448
H	-4.235502	3.768893	-1.339815
H	-4.135118	3.046504	1.056971
H	-3.450294	0.757012	1.640846

33

TS-4-5 BS4 el energy= -908.472151908

Mn	0.496954	-0.040245	-0.133770
O	-1.591877	2.080159	-0.082352
O	1.555974	1.012061	-2.680973
O	2.336178	1.744229	1.274867
C	1.647954	-1.896172	-0.632346
C	1.495791	-1.680762	0.734953
C	0.137105	-1.707261	1.349829
C	-0.974261	-2.542590	0.615997
C	-0.359924	-3.407565	-0.502582
C	0.597546	-2.639242	-1.431092
C	-2.125489	-1.621305	0.178256
C	-2.495504	-1.407855	-1.170072
C	-3.579012	-0.571482	-1.495730
C	-4.311797	0.059441	-0.485525
C	-3.966682	-0.156189	0.861346
C	-2.885743	-0.980721	1.186230
C	-0.798823	1.237257	-0.105411
C	1.134012	0.599221	-1.681370
C	1.605223	1.034618	0.713425
H	2.645713	-1.775999	-1.066680
H	2.356769	-1.437644	1.366049
H	0.193486	-1.879410	2.437948
H	-0.265632	-0.588609	1.418031
H	-1.389245	-3.228999	1.375216
H	0.209739	-4.226553	-0.031619
H	-1.165970	-3.879004	-1.087336
H	1.123429	-3.332365	-2.112552
H	0.051514	-1.956616	-2.113234
H	-1.981525	-1.928783	-1.981102
H	-3.851185	-0.429142	-2.545301
H	-5.158038	0.703641	-0.738839
H	-4.552334	0.309995	1.658665
H	-2.647532	-1.159707	2.241416
